# Monochromatic light reprograms transcription, metabolism, and rhizosphere microbial communities in *Salvia miltiorrhiza*

**DOI:** 10.1080/15592324.2026.2686334

**Published:** 2026-06-15

**Authors:** Xiaoyan Chen, Shuxian Ding, Huan Tang, Qing Yang, Langxing Yuan, Aling Zhang, Yingying Li, Qinglong Wang, Xiaoxia Yan, Zhunian Wang, Maoyuan Wang, Zai Zheng

**Affiliations:** a Tropical Crops Genetic Resources Institute, Chinese Academy of Tropical Agricultural Sciences/Identification and Evaluation Center for Tropical Agricultural Wild Plant Gene Resources, Ministry of Agriculture/Key Laboratory of Biology and Cultivation of Chinese Medicinal Materials, Ministry of Agriculture and Rural Affairs/ Hainan Engineering Research Center for Tropical Medicinal Plants, Haikou, China; b Sanya Research Institute, Chinese Academy of Tropical Agricultural Sciences, Sanya, China

**Keywords:** *Salvia miltiorrhiza*, monochromatic light, multi-omics, rhizosphere microbiome, tanshinones

## Abstract

*Salvia miltiorrhiza* is a valuable medicinal plant with diverse pharmacological applications and high market demand. Light quality is a critical environmental factor regulating plant growth, secondary metabolism, and interactions with rhizosphere microorganisms. However, the effects of short-term, pure monochromatic light exposure on *S. miltiorrhiza* remain largely unexplored. In this study, we employed integrated transcriptomic, metabolomic, and rhizosphere metagenomic analyzes to investigate the responses of *S. miltiorrhiza* under different monochromatic light conditions: ultraviolet (UV), blue (B), red (R), and far-red (FR), with white light (WL) as the control. GO enrichment analysis indicated that all monochromatic light treatments activated defense responses, while specific pathways related to light stimulus, wounding, and reactive oxygen species were uniquely enriched under B, R, and FR light. Metabolomic analysis showed a general decrease in metabolite abundance under monochromatic light compared to WL, with the R treatment inducing the highest number of significantly upregulated metabolites. Integrated KEGG pathway analysis of differential transcripts and metabolites highlighted the enrichment of secondary metabolic pathways, including diterpenoid, monoterpenoid, and phenylpropanoid biosynthesis. Notably, quantitative HPLC analysis confirmed that UV, R, and FR light significantly promoted the accumulation of dihydrotanshinone I and tanshinone IIA, while decreasing salvianolic acid A content. Metagenomic analysis revealed that monochromatic light, especially B light, reduced rhizosphere microbial alpha diversity and altered the abundance of specific bacterial families and species. Functional gene annotation also showed treatment-specific shifts in microbial metabolic potential and virulence factors. In conclusion, short-term monochromatic light culture, particularly R and FR, effectively modulates the transcriptome and metabolome of *S. miltiorrhiza*, enhancing the accumulation of key bioactive tanshinones, while simultaneously reshaping its rhizosphere microbial community. These findings offer a potential light-based strategy for improving the quality of *S. miltiorrhiza*.

## Introduction

*Salvia miltiorrhiza* Bunge is a perennial herb of the Lamiaceae family, widely recognized as a cornerstone of traditional Chinese medicine.^1^ Its dried roots are extensively used for the treatment of cardiovascular and cerebrovascular diseases, hepatic fibrosis, and chronic renal failure due to its rich content of bioactive compounds, including lipophilic tanshinones (e.g., tanshinone IIA, dihydrotanshinone I) and hydrophilic phenolic acids (e.g., salvianolic acid A).^2^ ^,^ ^3^ The increasing global demand for Danshen-based pharmaceuticals necessitates high-quality raw material, making optimized cultivation practices a research priority.

Light is a fundamental environmental signal that drives plant morphogenesis, photosynthesis, and secondary metabolism. Beyond its role in energy provision, light quality (spectral composition) is perceived by specific photoreceptors, such as phytochromes (red/far-red light receptors), cryptochromes and phototropins (blue/UV-A receptors), and UV-B receptors, which trigger complex intracellular signaling cascades that regulate gene expression and metabolic flux.^4-6^ Previous studies have demonstrated that supplemental red or blue light under a white light background can enhance plant growth, increase biomass, and improve tolerance to abiotic and biotic stresses in various species, including medicinal plants.^7^ ^,^ ^8^ For example, red light has been shown to promote the accumulation of anthocyanins and phenolic compounds, while blue light often enhances chlorophyll content and stomatal opening.^9^

The rhizosphere microbiome, the complex microbial community inhabiting the soil surrounding plant roots, plays an indispensable role in plant health and productivity. These microorganisms influence nutrient acquisition (nitrogen, phosphorus), produce phytohormones, antagonize pathogens, and prime the plant's systemic defense responses.^10^ ^,^ ^11^ Importantly, the composition and function of the rhizosphere microbiome are not static; they are dynamically shaped by the host plant's physiological state, which is, in turn, strongly influenced by environmental conditions such as light quality.^12^ ^,^ ^13^ Changes in light quality can alter root exudation patterns—the release of organic acids, sugars, and secondary metabolites—thereby selectively enriching or suppressing specific microbial taxa.^14^ ^,^ ^15^ Metagenomics has emerged as a powerful, culture-independent approach to comprehensively characterize the taxonomic composition, functional gene potential (e.g., virulence factors, carbohydrate-active enzymes), and metabolic capabilities of the rhizosphere microbiome.^16^ ^,^ ^17^

Despite these advancements, most studies have focused on the effects of supplemental monochromatic light within a full-spectrum (white light) background, or on the long-term acclimation of plants to different light qualities.^18-23^ The impact of short-term, pure monochromatic light culture—where plants are grown exclusively under a single wavelength band-on plant physiology and its rhizosphere partners is rarely reported.^24^ Furthermore, the integrated response of *S. miltiorrhiza* across the transcriptome, metabolome, and rhizosphere microbiome to different monochromatic lights (UV, B, R, FR) has not been systematically investigated. Understanding these holistic responses is crucial for developing novel light-based cultivation strategies to improve the yield and quality of medicinal plants.

Therefore, the objective of this study was to investigate the effects of three weeks of pure monochromatic light culture (UV, B, R, FR) on *S. miltiorrhiza*, using white light (WL) as a control. We employed an integrated multi-omics approach, combining transcriptomics, metabolomics, and rhizosphere metagenomics, to: (1) characterize the molecular and metabolic responses of *S. miltiorrhiza* to each monochromatic light, (2) quantify the accumulation of key bioactive compounds (salvianolic acid A, dihydrotanshinone I, tanshinone IIA), and (3) elucidate the concomitant changes in the taxonomic composition, diversity, and functional gene repertoire of the root-associated microbial community.

## Materials and methods

### Plant materials and growth conditions

Seeds of *Salvia miltiorrhiza* were germinated in seedling trays filled with a sterilized mixture of peat moss and vermiculite in a growth chamber under controlled conditions: 25 ± 1 °C, 60% relative humidity, and a 16 h photoperiod for five weeks. Uniform, healthy seedlings were then transplanted individually into pots, the seedlings were allowed to acclimate under the same white light conditions for one week before light treatments.

### Light treatments

Five light treatments were applied for three weeks in dedicated light-tight growth chambers (P50, CSNJ, Shenzhen, China) with controlled temperature (25 ± 1 °C), humidity (60%), and 16 h photoperiod. The light sources were LEDs with specific spectral peaks: White light (WL, control): full spectrum (400–700 nm). Ultraviolet light (UV): 380 nm. Blue light (B): 460 nm. Red light (R): 660 nm. Far-red light (FR): 730 nm. For UV, we used 30 µmol m⁻² s⁻¹; for B, R, and FR, we used 50 µmol m⁻² s⁻¹. Three biological replicates were used per treatment. After three weeks, whole seedlings were harvested for transcriptomic and metabolomic analyzes, and rhizosphere soil was collected for metagenomic analysis. All samples were immediately frozen in liquid nitrogen and stored at −80 °C.

### Transcriptome sequencing and analysis

Total RNA was extracted from *S. miltiorrhiza* seedlings using the RNAprep Pure Plant Kit (TIANGEN, Beijing, China) following the manufacturer's protocol. RNA integrity and purity were assessed by agarose gel electrophoresis and a NanoDrop spectrophotometer (Thermo Fisher Scientific, USA). mRNA was enriched using Oligo(dT) beads, fragmented, and used for cDNA library construction. Paired-end sequencing (150 bp) was performed on the DNBSEQ platform at Genepioneer Co., Ltd. (Nanjing, China). Raw sequencing reads were filtered to obtain clean reads, which were then aligned to the *S. miltiorrhiza* reference genome.^25^ Gene expression levels were quantified, and differentially expressed genes (DEGs) between sample groups were identified. Functional annotation and enrichment analyzes of DEGs were conducted using the Gene Ontology (GO) and Kyoto Encyclopedia of Genes and Genomes (KEGG) databases with the ClusterProfiler package in R and TBTools.^26^ ^,^ ^27^

### Metabolome analysis

Sample preparation and extraction followed established protocols.^28^ Metabolites were extracted from frozen tissue using 1 mL of 70% aqueous methanol via sonication for 30 min at 4 °C. Metabolite profiling was performed using an ultra-performance liquid chromatography (UPLC) system (Waters, USA) coupled to a Q Exactive high-resolution mass spectrometer (Thermo Fisher Scientific, USA). Chromatographic separation was achieved on a C^18^ column using a gradient elution with water (0.1% formic acid) and acetonitrile (0.1% formic acid). Mass spectrometry data were acquired in both positive and negative ion modes. Metabolites were identified by comparing mass spectra and retention times with authentic standards or against the mzCloud and public databases. Differential expressed metabolites (DEMs) were defined using the criteria VIP ≥ 1 (OPLS-DA) and |log_2_(fold change)|≥1. KEGG pathway enrichment analysis was performed on DEMs.^29^

### Quantification of tanshinones and salvianolic acid A by HPLC

For targeted quantification, 0.5 g of dried *S. miltiorrhiza* root powder was extracted with 25 mL of 75% methanol by ultrasonication for 45 min. The extract was filtered and analyzed using a HPLC system (Agilent 1260 Infinity II) equipped with a C^18^ column. The mobile phase was a gradient of water (A) and acetonitrile (B) at a flow rate of 1.0 mL/min and detection wavelength of 270 nm. Standard curves were constructed using authentic standards of salvianolic acid A, dihydrotanshinone I, and tanshinone IIA (Sigma-Aldrich, St. Louis, MO, USA). Three biological replicates were used per treatment.

### Rhizosphere soil metagenomic sequencing and analysis

Rhizosphere soil was collected by gently shaking the roots to remove loosely adhered soil, and the soil tightly attached to the root surface was collected using a sterile brush. Total DNA was extracted from rhizosphere soil using the DNeasy PowerSoil Pro Kit (Qiagen, Hilden, Germany) according to the manufacturer's instructions. DNA quality and quantity were assessed by agarose gel electrophoresis and a Qubit fluorometer (Thermo Fisher Scientific). Libraries were constructed using the Nextera DNA Flex Library Prep Kit (Illumina) and sequenced on the Illumina NovaSeq 6000 platform in Genepioneer Co., Ltd. (Nanjing, China).

Raw reads were quality-filtered using Trimmomatic (v0.39) and host (plant) reads were removed by mapping to the *S. miltiorrhiza* genome using Bowtie2 (v2.4.2). Clean reads were assembled using MEGAHIT (v1.2.9). Gene prediction was performed on assembled contigs (>500 bp) using Prodigal (v2.6.3). Nonredundant gene catalogs were constructed using CD-HIT (v4.8.1) with 95% identity and 90% coverage. Gene abundance was quantified using Salmon (v1.5.2) by mapping clean reads to the nonredundant gene set. Taxonomic classification was performed using Kraken2 (v2.1.1) against the NCBI RefSeq database. Functional annotation was carried out using: KEGG (KOBAS v3.0), KOG (eggNOG-mapper v2.0), VFDB (Virulence Factors Database, using Diamond), CARD (Comprehensive Antibiotic Resistance Database, using RGI v5.2.0), and CAZy (dbCAN2 v2.0). Alpha diversity indices (observed species, Chao1, Shannon) were calculated using QIIME2 (v2021.8). Linear discriminant analysis effect size (LEfSe) was used to identify differentially abundant taxa (LDA score > 2, *p* < 0.05).

### Statistical analysis

All experiments were performed with three independent biological replicates. Data are presented as means ± SE. Statistical significance between two groups was assessed using unpaired Student's t-test. For multigroup comparisons, one-way ANOVA followed by Tukey's HSD post hoc test was used. A *p*-value < 0.05 was considered statistically significant. All statistical analyzes were performed using R software (v4.1.2).

## Results

### Phenotypic and transcriptomic responses to monochromatic light

**Figure 1.**
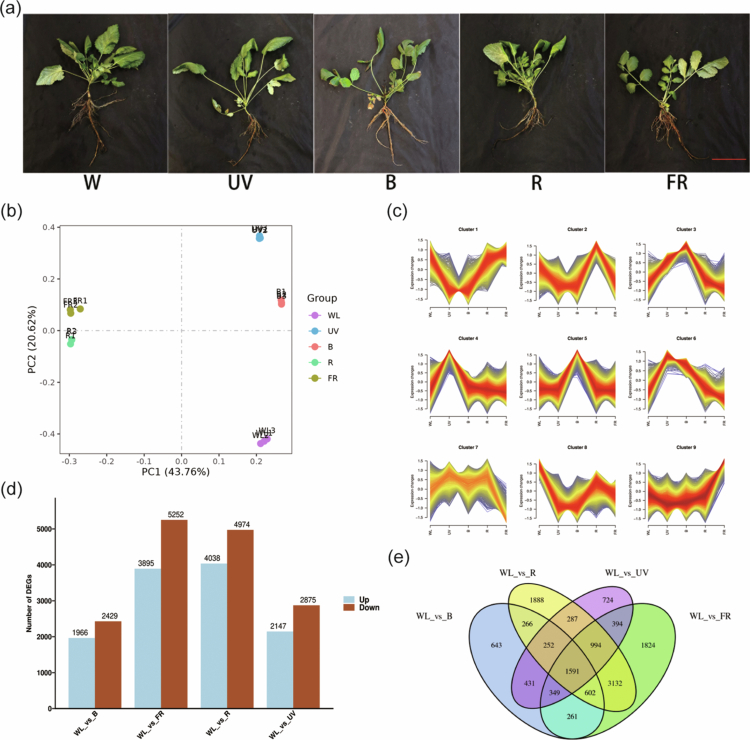
Phenotypic and transcriptomic analysis of *Salvia miltiorrhiza* under different monochromatic light conditions. (a) Phenotype of *S. miltiorrhiza* after three weeks of exposure to white light (WL), ultraviolet light (UV), blue light (b), red light (R), and far-red light (FR). Scale bar = 10 cm. (B) Principal component analysis (PCA) of transcriptome data from the different treatment groups (*n* = 3). (c) Expression trend clusters of genes across the different treatment groups. Nine distinct clusters are shown. (d) Number of differentially expressed genes (DEGs) in each treatment group compared to WL (|log_2_FC|≥1, FDR < 0.05). Upregulated (blue) and downregulated (brown) genes are shown. (e) Venn diagram showing the overlap of DEGs among the WL_UV, WL_B, WL_R, and WL_FR comparisons.

After three weeks of exposure to different monochromatic light conditions (WL, UV, B, R, FR), *S. miltiorrhiza* plants did not show any striking morphological differences compared to the WL control (Figure 1a). However, transcriptomic analysis revealed profound molecular reprogramming. Principal component analysis (PCA) of the transcriptomes showed clear separation among the five treatment groups, with tight clustering of biological replicates within each group, indicating high reproducibility and effective treatment-induced transcriptomic shifts (Figure 1b). This high intra-group reproducibility was further confirmed by heatmap visualization of all differentially expressed genes (DEGs), which showed consistent expression patterns among replicates of the same treatment (Supplemental Figure 1). To visualize global expression patterns, genes were clustered into nine distinct clusters based on their expression trends across treatments (Figure 1c). Notably, each monochromatic light treatment exhibited a specific cluster where gene expression was uniquely elevated compared to all other groups, suggesting specialized transcriptional responses.

The number of DEGs varied substantially among treatments (Figure 1d). Compared to WL, the WL_FR comparison yielded the highest number of DEGs (3895 upregulated, 5252 downregulated), followed by WL_R (4038 up, 4974 down), WL_UV (2147 up, 2875 down), and WL_B (1966 up, 2429 down) (Supplemental Table 1). A Venn diagram of DEGs across the four pairwise comparisons (WL_UV, WL_B, WL_R, WL_FR) revealed that each treatment induced a substantial number of unique DEGs: WL_UV had 724 unique DEGs, WL_B had 643, WL_R had 1888, and WL_FR had 1824 (Figure 1e). These results indicate that different monochromatic lights trigger distinct and largely nonoverlapping transcriptional response mechanisms in *S. miltiorrhiza*.

### Functional enrichment of differentially expressed genes

**Figure 2.**
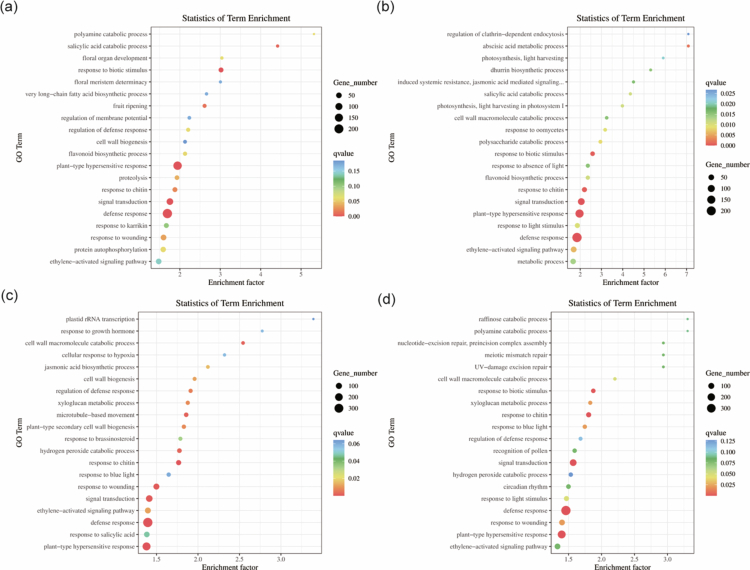
GO (biological process) analysis of DEGs in different treatment groups. (a) WL_UV, (b) WL_B, (c) WL_R, (d) WL_FR. Only the top 10 most significantly enriched terms (by q-value) are shown for each group. Dot size represents the number of DEGs, and color represents the q-value.

To understand the biological processes affected, Gene Ontology (GO, Biological Process) enrichment analysis was performed on the DEGs from each comparison (Figure 2). In the WL_UV group, the most significantly enriched terms were “defense response”, “signal transduction”, “plant-type hypersensitive response”, “response to biotic stimulus”, and “salicylic acid catabolic process” (Figure 2a). This suggests that UV light activates plant defense responses and hormone signaling. In the WL_B group, similar defense‑related terms were enriched, but additionally, “photosynthesis,” “response to absence of light,” and “response to light stimulus” appeared (Figure 2b), indicating that blue light specifically induces genes related to light perception and photosynthetic acclimation. In the WL_R group, besides the common defense terms, “response to wounding” and “hydrogen peroxide catabolic process” were significantly enriched (Figure 2c). This implies that red light may regulate wounding‑related pathways and reactive oxygen species (ROS) homeostasis. Interestingly, “response to blue light” was also enriched, potentially reflecting a compensatory response to the absence of blue light. In the WL_FR group, the enriched terms included “response to light stimulus,” “response to blue light,” and “UV‑damage excision repair” alongside core defense responses (Figure 2d), indicating that far‑red light broadly impacts light‑associated regulatory systems and DNA repair mechanisms.

Complementing the GO analysis, KEGG pathway enrichment of the same DEG sets (Supplemental Figure 2) revealed that “plant‑pathogen interaction” was a common enriched pathway across all monochromatic light treatments, further supporting the induction of defense‑related signaling. Treatment‑specific KEGG pathways were also identified: “diterpenoid biosynthesis” was enriched in both WL_UV and WL_B groups (Supplemental Figure 2a and b); “zeatin biosynthesis” was uniquely enriched in the WL_R group (Supplemental Figure 2c); and the “MAPK signaling pathway” was specifically enriched in the WL_FR group (Supplemental Figure 2d). These pathway‑level differences highlight the wavelength‑specific regulatory programs activated by each monochromatic light.

### Metabolomic reprogramming under monochromatic light

**Figure 3.**
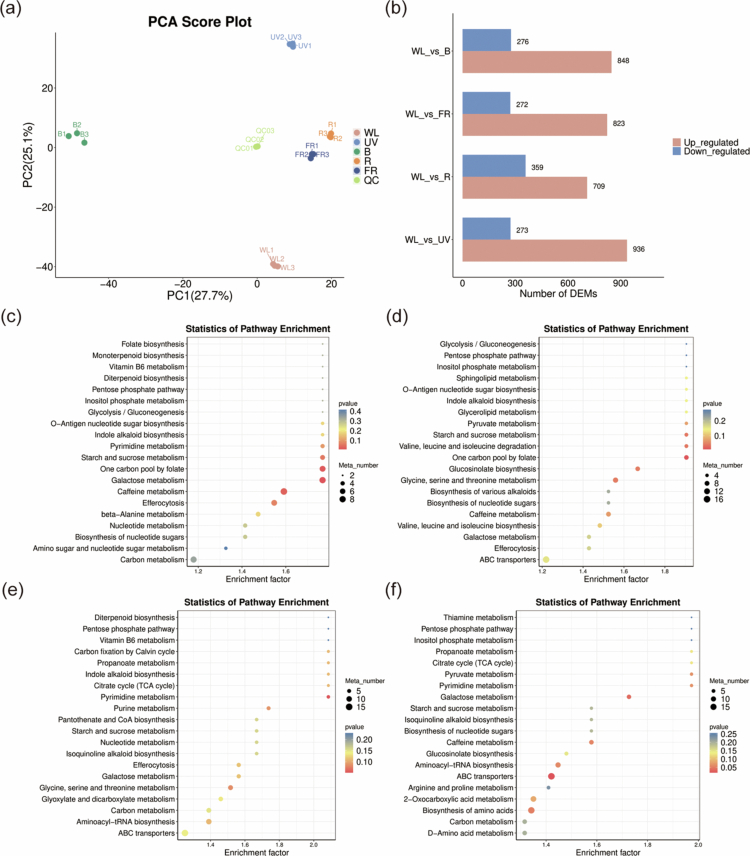
Metabolomic analysis under different monochromatic light conditions. (a) Principal component analysis (PCA) of metabolomics data across different treatment groups (*n* = 3). (b) Number of differentially expressed metabolites (DEMs) in each treatment group compared to WL (VIP ≥ 1, |log_2_FC|≥1). Upregulated (blue) and downregulated (brown) genes are shown. KEGG pathway enrichment analysis of DEMs in the (c) WL_UV, (d) WL_B, (e) WL_R, and (f) WL_FR groups. Only the top 10 most significantly enriched pathways (by *p*-value) are shown for each group.

To complement the transcriptomic data, we performed untargeted metabolomics. PCA of the metabolomics data showed clear separation among the five groups with good intragroup reproducibility (Figure 3a). Notably, the R and FR groups were positioned closer to each other, consistent with the physical similarity of these two light qualities. Heatmap visualization of all differentially expressed metabolites (DEMs) confirmed the clear separation between treatment groups and the consistency among biological replicates (Supplemental Figure 3).

The number of DEMs varied across treatments (Figure 3b). All monochromatic light treatments resulted in a general decrease in metabolite abundance compared to WL (WL_UV: 273 up, 936 down; WL_B: 278 up, 848 down; WL_R: 359 up, 709 down; WL_FR: 272 up, 823 down) (Supplemental Table 2). The R treatment induced the highest number of significantly upregulated metabolites (359), suggesting a stronger stimulatory effect on certain metabolic pathways.

A Venn diagram of DEMs (Supplemental Figure 4) showed that all treatment groups shared 536 common DEMs, indicating a core set of metabolites responsive to monochromatic light irrespective of wavelength. Each treatment also possessed a modest number of unique DEMs: 78 for WL_UV, 82 for WL_B, 78 for WL_R, and 46 for WL_FR. The specific metabolites that were significantly upregulated or downregulated in each treatment are listed in Supplemental Figure 5. For example, silibinin B was commonly upregulated in UV, B, and FR treatments, while angoroside A and verbascoside A were consistently downregulated across multiple treatments (Supplemental Figure 5).

KEGG pathway enrichment of DEMs revealed treatment‑specific patterns (Figure 3). For WL_UV, enriched pathways included “caffeine metabolism”, “galactose metabolism”, “one carbon pool by folate”, and “starch and sucrose metabolism” (Figure 3c), which are often associated with stress responses. For WL_B, pathways such as “glycine, serine and threonine metabolism”, and “valine, leucine and isoleucine degradation” were prominent (Figure 3d), indicating a role for blue light in regulating amino acid metabolism. For WL_R, “glycine, serine and threonine metabolism”, “purine metabolism”, and “pyrimidine metabolism” were enriched (Figure 3e). For WL_FR, the most significant enrichment was “ABC transporters” along with “biosynthesis of amino acids” and “aminoacyl‑tRNA biosynthesis” (Figure 3f), suggesting that far‑red light uniquely modulates cellular transport and protein synthesis machinery.

### Integrated analysis and accumulation of bioactive compounds

**Figure 4.**
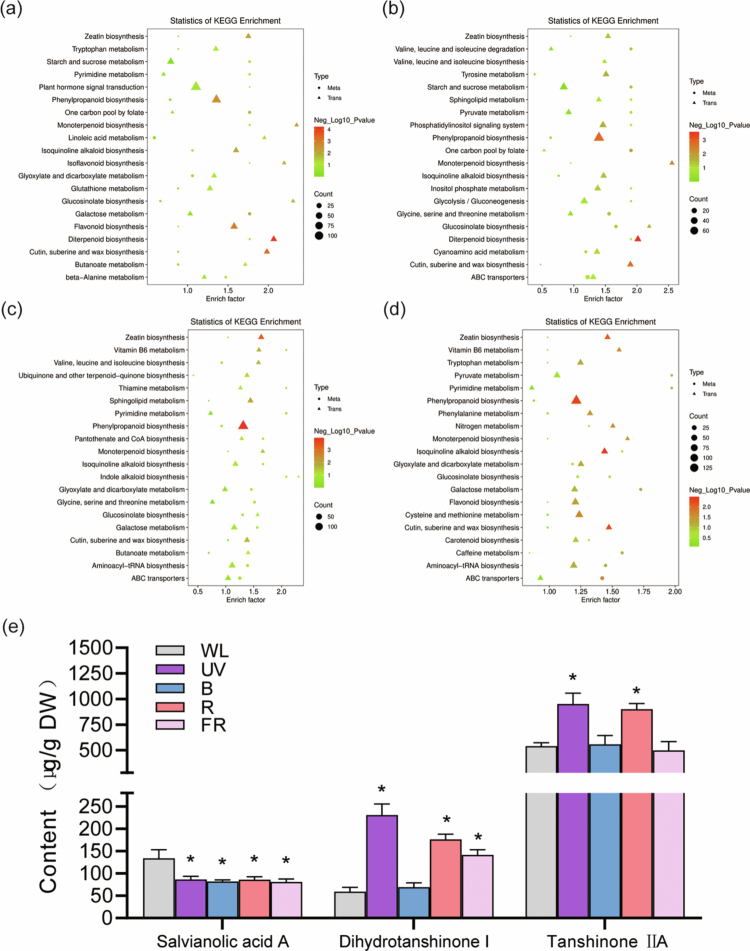
UV, red, and far-red light promote the accumulation of tanshinones. Integrated KEGG pathway analysis of differential transcriptomic and metabolomic data in the (a) WL_UV, (b) WL_B, (c) WL_R, and (d) WL_FR group. The x-axis represents the enrich factor, and the y-axis represents the pathway name. Dot size indicates the number of differential genes/metabolites in the pathway, and color indicates the *p*-value. (e) Quantification of Salvianolic acid A, Dihydrotanshinone I, and Tanshinone IIA in *S. miltiorrhiza* roots under different light conditions. Data are presented as means ± SE (*n* = 3). Significant differences compared to the WL control were analyzed using unpaired t-test: *p < 0.05.

Integrated KEGG pathway analysis of combined differential transcriptomic and metabolomic data was performed to identify co‑regulated pathways. For WL_UV, pathways such as “diterpenoid biosynthesis”, “monoterpenoid biosynthesis”, and “isoflavonoid biosynthesis” were significantly enriched (Figure 4a). WL_B also showed enrichment in “diterpenoid biosynthesis” and “monoterpenoid biosynthesis” (Figure 4b). WL_R uniquely enriched “zeatin biosynthesis”, “vitamin B6 metabolism”, and “indole alkaloid biosynthesis” (Figure 4c). WL_FR enriched “cutin, suberine and wax biosynthesis”, “isoquinoline alkaloid biosynthesis”, “nitrogen metabolism”, and notably, “ABC transporters” (Figure 4d), consistent with the metabolomic findings.

Because several pathways related to secondary metabolite biosynthesis (e.g., diterpenoid, phenylpropanoid, flavonoid) were consistently enriched, we hypothesized that monochromatic light affects the accumulation of key bioactive compounds in *S. miltiorrhiza*. We quantified three representative compounds by HPLC (Figure 4e). Compared to WL, all monochromatic light treatments significantly reduced the content of salvianolic acid A. However, UV, R, and FR light significantly increased dihydrotanshinone I content by 2–4 fold. For tanshinone IIA, UV and R light significantly enhanced its accumulation. These results demonstrate that monochromatic light culture, particularly with UV, R, or FR, can be an effective strategy to improve the quality of *S. miltiorrhiza* by promoting the accumulation of lipophilic tanshinones, albeit at the cost of reducing hydrophilic phenolic acids.

### Metagenomic analysis of rhizosphere microbial communities

**Figure 5.**
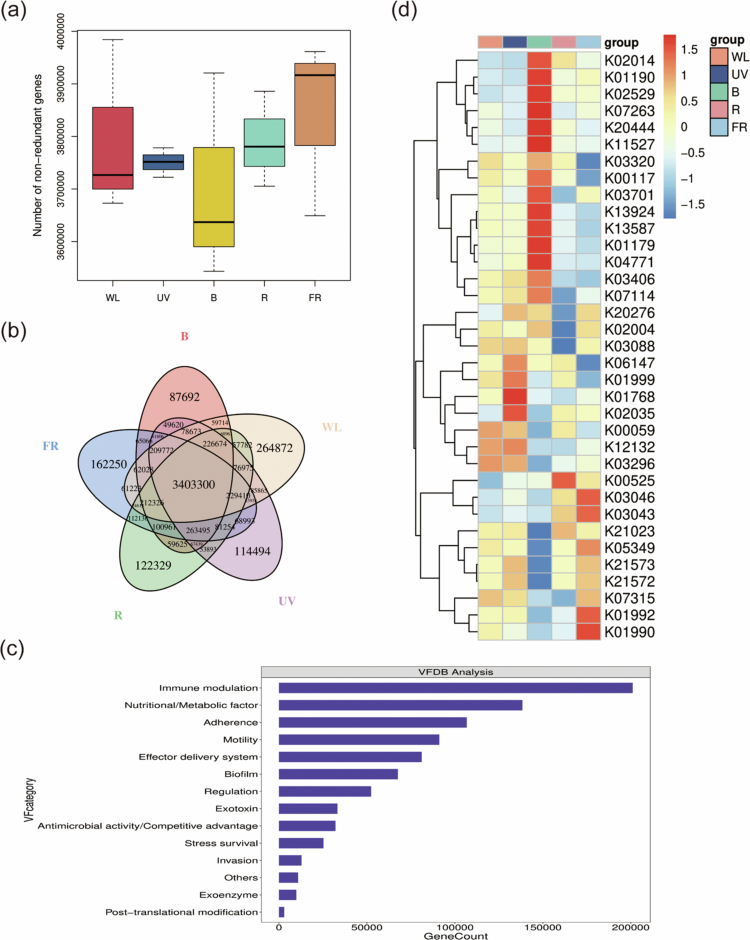
Metagenomic analysis under different monochromatic light conditions. (a) Number of metagenomic sequencing genes (nonredundant gene catalog) among the different treatment groups. (b) Venn diagram showing the overlap of metagenomic sequencing genes among the different treatment groups. (c) VFDB analysis of metagenomic sequencing gene functions across different treatment groups, showing the number of genes associated with each virulence factor category. (d) KOG analysis of metagenomic sequencing genes across different treatment groups. Only selected KOG categories with significant differences are shown.

Metagenomic sample reproducibility was first assessed by correlation analysis, which showed high intra‑group correlations, confirming the reliability of the sequencing data (Supplemental Figure 6). The number of metagenomic sequencing genes was similar among all treatment groups, with the B group showing a slightly lower count (Figure 5a). A Venn diagram of all genes revealed a large core set of 3,403,300 genes shared across all treatments, indicating a stable core rhizosphere microbiome (Figure 5b). However, the WL treatment had the highest number of unique genes, while each monochromatic light treatment, especially B, had fewer unique genes, suggesting that monochromatic light reduces the genetic novelty or rare biosphere in the rhizosphere. Furthermore, VFDB analysis showed that the most abundant virulence factor categories across all treatments were “immune modulation”, “nutritional/metabolic factor”, “adherence”, “motility”, and “effector delivery system,” which are critical for microbial survival, colonization, and host interactions (Figure 5c).

KOG analysis of the metagenomic genes revealed treatment‑specific functional shifts (Figure 5d). For example, Compared to WL, the UV treatment significantly increased the abundance of genes associated with KOG categories K06147, K01999, K01768, and K02035. The B treatment induced the most changes, with significant upregulation of 12 KOGs (e.g., K02014, K01190, K02529) and downregulation of 7 KOGs (e.g., K21023, K05349). R treatment downregulated K07114, K20276, K02004, K03088, K07315, K03701, and upregulated K00525, K03046, K03043. FR treatment upregulated K01990, K01992, K21572, K21573, K05349, K03046, K03043, and downregulated K00059, K01999, K06147, K03406, K13587, K00117, K03320 (Figure 5d). A broader COG functional classification (Supplemental Figure 7) showed that “amino acid transport and metabolism” was the most abundant category across all treatments in terms of both gene count and relative abundance, followed by “carbohydrate transport and metabolism” and “transcription”.

To assess the potential for antibiotic resistance and carbohydrate degradation, we performed CARD and CAZy analyzes. CARD analysis revealed no significant difference in the total number of detected antibiotic resistance genes (ARGs) among treatments (Supplemental Figure 8a). A Venn diagram of ARGs (Supplemental Figure 8b) showed that all groups shared 31 common ARGs, while only the B treatment possessed one unique ARG. Notably, the relative abundance of the adeF ARG was significantly higher in the B treatment compared to all other groups (Supplemental Figure 8c). A heatmap of all detected ARGs showed distinct clustering of the B treatment (Supplemental Figure 8D). CAZy analysis (Supplemental Figure 9) showed that glycoside hydrolases (GH) were the most abundant carbohydrate‑active enzyme class across all treatments in terms of gene count. In terms of relative abundance, the B treatment had a higher proportion of GH and glycosyl transferase (GT) genes compared to the other groups (Supplemental Figure 9b), suggesting that blue light may enrich for microbes with enhanced capabilities for carbohydrate degradation and transfer.

### Microbial diversity and taxonomic composition

**Figure 6.**
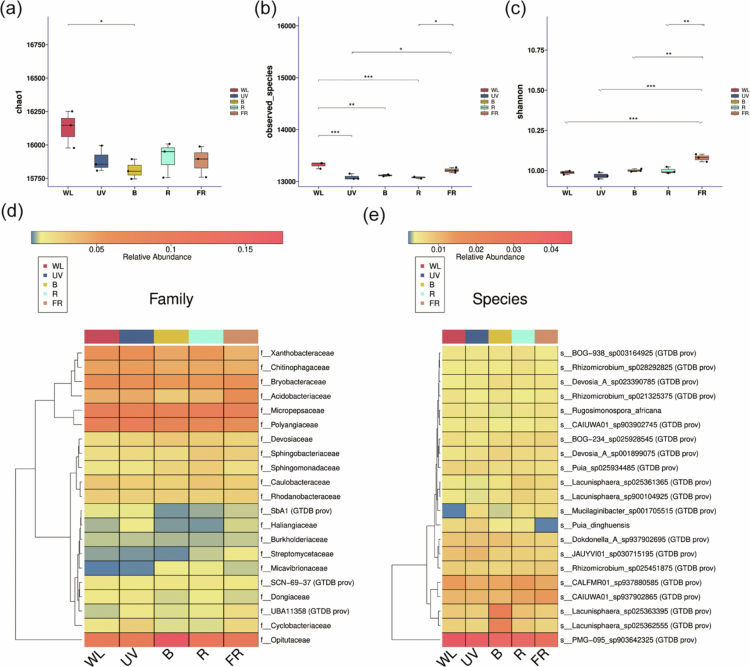
Analysis of microbial community diversity and classification under different monochromatic light conditions. (a) Observed species index, (b) Chao1 index, and (c) Shannon index of metagenomic microbial communities. Data are presented as means ± SE (*n* = 3). Significant differences between each monochromatic light treatment and the WL control are indicated by *p < 0.05, **p < 0.01, ***p < 0.001. (d) Heatmap showing the relative abundance of differentially abundant microbial families across treatments. (e) Heatmap showing the relative abundance of differentially abundant microbial species across treatments. Color scale represents relative abundance.

Alpha diversity analysis revealed that monochromatic light generally reduced rhizosphere microbial richness. The observed species index was significantly lower in UV, B, and R treatments compared to WL (Figure 6a). The Chao1 index, which estimates total species richness, was decreased in all monochromatic treatments, with the B treatment showing a statistically significant reduction (Figure 6b), suggesting that blue light may cause the disappearance or inhibition of many microbial species. Shannon index analysis showed that the FR treatment had significantly different diversity compared to all other treatments (Figure 6c), indicating that far‑red light alters overall community evenness or richness, potentially by increasing certain rare taxa.

To identify specific microbial taxa that respond to light quality, we performed LEfSe analysis. This analysis revealed potential microbial biomarkers for each light condition: *f_Xanthobacteraceae* (family) for WL, *f_Chitinophagales* (family) for UV, *p_Pseudomonatota* (phylum) for B, *c_Alphaproteobacteria* (class) for R, and *c_Actinomycetota* (class) for FR (Supplemental Figure 10). These biomarkers may serve as indicators of light‑quality effects on the rhizosphere.

Heatmaps of family‑level and species‑level differential abundance further revealed specific taxa responding to light treatments (Figure 6d and e). At the family level, *Haliangiaceae* was significantly enriched under UV; *Micavibrionaceae* was enriched under B and FR; and sbA1 (GTDB prov) was significantly reduced under B and R. At the species level, *Mucilaginibacter_sp001705515* was significantly increased under all monochromatic lights (UV, B, R, FR), while *Puia_dinghuensis* was significantly decreased under FR (Figure 6e).

## Discussion

In this study, we systematically investigated the multi-omic responses of *Salvia miltiorrhiza* to short-term pure monochromatic light culture (UV, B, R, FR) compared to WL. While no overt phenotypic changes were observed, our integrated transcriptomic, metabolomic, and metagenomic analyzes revealed profound molecular, biochemical, and ecological reprogramming. This suggests that plants perceive monochromatic light as a significant environmental signal and mount complex adaptive responses that are not immediately visible at the macroscopic level. Our results provide the first holistic view of how light quality simultaneously shapes plant secondary metabolism and its associated rhizosphere microbiome.

A striking and consistent finding across all monochromatic light treatments was the strong enrichment of DEGs involved in “defense response,” “plant-type hypersensitive response,” and “signal transduction” (Figure 2). This indicates that growth under pure monochromatic light, irrespective of the specific wavelength, may be perceived as a stressor by *S. miltiorrhiza*. This is plausible because under natural conditions, plants are exposed to a full solar spectrum. The absence of certain wavelengths or the excess of a specific band likely disrupts the normal photoperceptive equilibrium, triggering a generalized stress response.^30^ To explore the underlying mechanisms, we performed GSEA on the WL_UV group and found significant enrichment of the MAPK signaling pathway, plant-pathogen interaction pathway, and flavonoid and terpenoid biosynthesis pathways (Supplemental Figure 11). Activation of the MAPK cascade is a well-known early signaling event in plant stress responses, linking external stimuli to downstream defense gene expression. The concurrent enrichment of “salicylic acid catabolic process” in UV (Figure 2a) and “hydrogen peroxide catabolic process” in R (Figure 2c) further supports the activation of hormonal and redox stress signaling pathways. Specifically, our metabolomic data revealed significant accumulation of salicylic acid (SA), ACC (a precursor of ethylene), and IAA under UV treatment (Supplemental Figure 12), suggesting crosstalk between defense-related hormones and growth regulators. These observations are consistent with previous reports that light stress can mimic pathogen attack and prime plant immunity.^31^ ^,^ ^32^

Beyond the core defense response, each monochromatic light induced unique transcriptional and metabolic signatures. Blue light uniquely enriched “photosynthesis” and “response to light stimulus” genes (Figure 2b), reflecting the role of cryptochromes in optimizing photosynthetic apparatus under blue-deficient conditions.^33^ Red light uniquely enriched “response to wounding” (Figure 2c), suggesting a link between phytochrome signaling and mechanical damage responses, possibly via jasmonic acid pathways.^34^ ^,^ ^35^ Far-red light, which is enriched in shade conditions, induced a broad “response to light stimulus” and “UV-damage excision repair” (Figure 2d), indicating that FR light might activate a general photoprotective and DNA repair program, possibly through phytochrome A-mediated signaling.^36^

The metabolomic data showed that all monochromatic lights caused a net decrease in total metabolite abundance (Figure 3b), underscoring the importance of full-spectrum light for maintaining primary and secondary metabolic homeostasis. However, the R treatment was unique in inducing the highest number of upregulated metabolites (359). This aligns with the well-known role of red light in activating the expression of genes encoding enzymes in the phenylpropanoid and terpenoid biosynthetic pathways via phytochrome B.^37^ The integrated pathway analysis (Figure 4) further corroborated this, showing that R and FR light-enriched pathways leading to the biosynthesis of tanshinones (diterpenoids) and other secondary metabolites.

A more nuanced picture emerges when examining specific pathways. Using the WL_UV group as an example, we observed that key genes in the phenylpropanoid biosynthesis pathway were significantly affected (Supplemental Figure 13). Concomitantly, the levels of TCA cycle intermediates (citrate and isocitrate) increased, and the accumulation of coumarins, phenylpropanoids, and flavonoids was significantly altered (Supplemental Figure 14). The TCA cycle provides carbon skeletons and energy for secondary metabolism; thus, changes in TCA cycle intermediates may directly influence the flux into downstream pathways.

The most practically significant finding was the effect on bioactive compound accumulation (Figure 4E). While all monochromatic lights reduced salvianolic acid A (a hydrophilic phenolic acid), UV, R, and FR significantly increased dihydrotanshinone I and tanshinone IIA (lipophilic diterpenoids). This differential effect on the two major classes of active compounds suggests a regulatory trade-off. Light quality might redirect carbon flux from the shikimate/phenylpropanoid pathway (leading to phenolic acids) toward the mevalonate/MEP pathways (leading to terpenoids).^38-40^ The specific induction of tanshinones under R and FR light is consistent with the enrichment of “diterpenoid biosynthesis” in our integrated analysis. This observation has practical implications: short-term exposure to R or FR light before harvest could serve as a simple, nonchemical treatment to boost the content of valuable tanshinones in *S. miltiorrhiza* roots, although this remains a potential strategy that requires further validation under field conditions.

Our metagenomic analysis revealed that monochromatic light also significantly reshapes the rhizosphere microbial community. The reduction in alpha diversity (observed species and Chao1) under monochromatic light, particularly B (Figure 6a and b), indicates that a full light spectrum promotes a more diverse microbial community. This is consistent with the hypothesis that a more diverse array of root exudates, produced under full-spectrum light, supports a more diverse microbiome.^41-44^ The unique effect of FR light on the Shannon index (Figure 6c) suggests that far-red light may favor a more even community, potentially by suppressing dominant taxa and allowing rare taxa to proliferate.

Functional metagenomic analysis provided clues about the metabolic potential of the shifted microbiome. The enrichment of specific KOG categories (Figure 5c) and the increased abundance of GH and GT genes under B light (Supplemental Figure 10) suggest that blue light may enrich for microbes with enhanced capabilities for carbohydrate degradation and transport. This could be a response to altered carbon availability in the rhizosphere. The increased abundance of the adeF ARG under B light (Supplemental Figure 9c) is intriguing, but its ecological significance (whether it confers a fitness advantage under B light or is a coincidental coselection) requires further investigation.^45^ ^,^ ^46^

Several limitations of this study should be explicitly acknowledged. First, the use of pure monochromatic light without a white light background represents a non-natural condition. While this design allowed us to isolate the effects of individual wavelengths, the observed responses may partly reflect stress-induced artifacts rather than normal physiological regulation. Second, our experiment was relatively short-term (three weeks), and we did not observe significant phenotypic changes or collect yield/biomass data. Whether the observed metabolic and microbial shifts are sustained over longer periods or translate into improved field performance remains unknown. Third, our conclusions about improved “quality” are based solely on tanshinone content; a comprehensive quality assessment would also include other bioactive compounds, sensory attributes, and safety parameters. Fourth, the proposed model linking root metabolites to microbiome shifts remains hypothetical and requires validation through targeted experiments, such as root exudate profiling combined with synthetic community (SynCom) assays. Future studies should also investigate whether light-recruited microbes can feedback to enhance plant growth or stress tolerance.

## Conclusions

**Figure 7.**
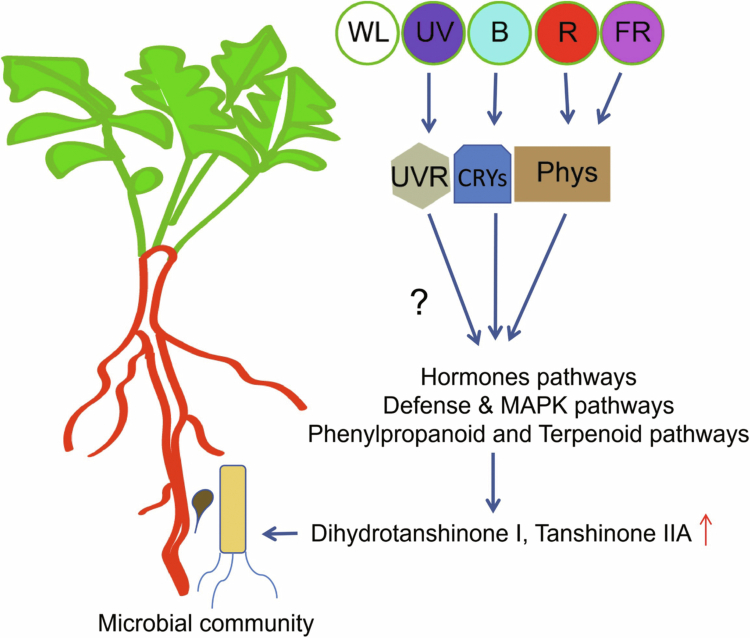
A proposed model for monochromatic light regulation of metabolism and the rhizosphere microbiome in *S. miltiorrhiza*. Irradiation with monochromatic light activates photoreceptors such as UVR, CRYs, and phytochromes (Phys), which subsequently trigger intracellular signaling cascades regulating multiple pathways, including hormone signaling, defense responses, and the biosynthesis of phenylpropanoids and terpenoids. These changes ultimately alter the metabolite profile in *S. miltiorrhiza* roots, thereby reshaping the composition of the rhizosphere microbial community.

Taken together, our results demonstrate a complex, wavelength-specific interaction among light, the plant host, and its rhizosphere microbiome. Based on these findings, we propose a working model (Figure 7). In this model, monochromatic light is perceived by photoreceptors such as UVR8, CRYs, and phytochromes (Phys). This perception activates downstream signaling cascades, including the MAPK pathway and hormone signaling (SA, ethylene, auxin), which in turn regulate unknown transcription factors (e.g., bZIP, MYB, WRKY) and the expression of genes involved in defense responses, phenylpropanoid biosynthesis, and terpenoid biosynthesis. These transcriptional and metabolic changes alter the root metabolite profile, including a trade-off between phenolic acids and tanshinones. Consequently, changes in root exudation patterns reshape the composition and functional potential of the rhizosphere microbial community. Conversely, changes in the microbial community might feed back on the plant, modulating its stress responses and secondary metabolism.^47-49^ This model provides a framework for future hypothesis-driven research into light-mediated regulation of plant-microbe interactions in medicinal plants.

## Supplementary Material

Supplemental Figures-clean.docx Supplemental Figures-clean.docx

Supplemental Table 1_DEGs summary.xls Supplemental Table 1_DEGs summary.xls

Supplemental Table 2_DEMs summary.xlsx Supplemental Table 2_DEMs summary.xlsx

## Data Availability

The original contributions presented in the study are included in the article/Supplemental Materials; further inquiries can be directed to the corresponding authors.

## References

[1] Li ZM , Xu SW , Liu PQ . *Salvia miltiorrhiza burge* (Danshen): a golden herbal Medicine in cardiovascular therapeutics. Acta Pharmacol Sin. 2018;39:802–824. doi: 10.1038/aps.2017.193. 29698387 PMC5943903

[2] Zhao H , Han B , Li X , Sun C , Zhai Y , Li M , Jiang M , Zhang W , Liang Y , Kai G . *Salvia miltiorrhiza* in breast cancer treatment: a review of its phytochemistry, derivatives, nanoparticles, and potential mechanisms. Front Pharmacol. 2022;13:872085. doi: 10.3389/fphar.2022.872085. 35600860 PMC9117704

[3] Heo JY , Im DS . Anti-allergic effects of salvianolic acid A and tanshinone IIA from *salvia miltiorrhiza* determined using in vivo and in vitro experiments. Int Immunopharmacol. 2019;67:69–77. doi: 10.1016/j.intimp.2018.12.010. 30537633

[4] Meng C , Joanne C , Christian F . Light signal transduction in higher plants. Annu Rev Genet. 2004;38:87–117. doi: 10.1146/annurev.genet.38.072902.092259. 15568973

[5] Kanegae T , Hayashida E , Kuramoto C , Wada MA . Single chromoprotein with triple chromophores acts as both a phytochrome and a phototropin. P NATL ACAD SCI USA. 2006;103:17997–18001. doi: 10.1073/pnas.0603569103. PMC1693861 17093054

[6] Jenkins GI , Long JC , Wade HK , Shenton MR , Bibikova TN . UV and blue light signalling: pathways regulating chalcone synthase gene expression in arabidopsis. New Phytol. 2001;151:121–131. doi: 10.1046/j.1469-8137.2001.00151.x. 33873370

[7] ierik R , Ballaré CL . C. L. control of plant growth and defense by photoreceptors: from mechanisms to opportunities in agriculture. Mol Plant. 2021;14:61–76. doi: 10.1016/j.molp.2020.11.021. 33276158

[8] Xu F , He S , Zhang J , Mao Z , Wang W , Li T , Hua J , Du S , Xu P , Li L , et al. Photoactivated CRY1 and phyB interact directly with AUX/IAA proteins to inhibit auxin signaling in arabidopsis. Mol Plant. 2018;11:523–541. doi: 10.1016/j.molp.2017.12.003. 29269022

[9] Zhu Y , Patil BS , Zhen S . From ultraviolet-B to red photons: effects of end-of-production supplemental light on anthocyanins, phenolics, ascorbic acid, and biomass production in red leaf lettuce. PLoS One. 2025;20:e0328303. doi: 10.1371/journal.pone.0328303. 40737257 PMC12309995

[10] Cucu MA , Terzi V , Fossalunga ASd , Carnevali P , Ghizzoni R , Morcia C . Cereal health regulation by arbuscular mycorrhizal fungi (AMF): insights from tripartite Plant–AMF–Pathogen systems within the one health framework. Agronomy. 2026;16:621. doi: 10.3390/agronomy16060621.

[11] Geemarahalli Mahadevaswamy S , De Britto S , Satapute P , Abdelrahman M , Tran LSP , Sasaki K , Amruthesh KN , Jogaiah S . Rhizosphere fungi mediated priming reinforces cellular and biochemical defenses against gummy stem blight in muskmelon. Rhizosphere. 2025;36:101236. doi: 10.1016/j.rhisph.2025.101236.

[12] Zhou Y , Coventry DR , Gupta VVSR , Fuentes D , Merchant A , Kaiser BN , Li J , Wei Y , Liu H , Wang Y , et al. The preceding root system drives the composition and function of the rhizosphere microbiome. Genome Biol. 2020;21:89. doi: 10.1186/s13059-020-01999-0. 32252812 PMC7137527

[13] Li Q , Lan Y , Yang Y , Kang S , Wang X , Jiang J , Liu S , Wang Q , Zhang W , Zhang L . Effect of luminescent materials on the biochemistry, ultrastructure, and rhizobial microbiota of spirodela polyrhiza. Plant Physiol Bioch. 2024;207:108427. doi: 10.1016/j.plaphy.2024.108427. 38367389

[14] Wiesenbauer J , Gorka S , Jenab K , Schuster R , Kumar N , Rottensteiner C , König A , Kraemer S , Inselsbacher E , Kaiser C . Preferential use of organic acids over sugars by soil microbes in simulated root exudation. Soil Biol Biochem. 2025;203:109738. doi: 10.1016/j.soilbio.2025.109738.

[15] Seitz VA , McGivern BB , Borton MA , Chaparro JM , Schipanski ME , Prenni JE , Wrighton KC . Cover crop root exudates impact soil microbiome functional trajectories in agricultural soils. Microbiome. 2024;12:183. doi: 10.1186/s40168-024-01886-x. 39342284 PMC11439266

[16] Prabha R , Singh DP , Gupta S , Gupta VK , El-Enshasy HA , Verma MK . Rhizosphere metagenomics of *paspalum scrobiculatum* L. (Kodo Millet) Reveals rhizobiome multifunctionalities. Microorganisms. 2019;7:608. doi: 10.3390/microorganisms7120608. 31771141 PMC6956225

[17] Alnahari AA , Alshehrei FM . Rhizospheric microbiomes as reservoirs for multifaceted agricultural, environmental, and industrial applications. Biomass Bioenerg. 2026;211:109118. doi: 10.1016/j.biombioe.2026.109118.

[18] Samkumar A , Karppinen K , McGhie TK , Espley RV , Martinussen I , Jaakola L . Flavonoid biosynthesis is differentially altered in detached and attached ripening bilberries in response to spectral light quality. Front Plant Sci. 2022;13. doi: 10.3389/fpls.2022.969934. PMC9355381 35937358

[19] Lozano-Castellanos LF , Pennisi G , Navas-Gracia LM , Orsini F , Sánchez-Hernández E , Martín-Ramos P , Correa-Guimaraes A . Physiological and phytochemical responses of calendula officinalis L. To end-of-day Red/Far-Red and Green light. Biology. 2025;14:935. doi: 10.3390/biology14080935. 40906072 PMC12383836

[20] Claypool NB , Lieth JH . Physiological responses of pepper seedlings to various ratios of blue, Green, and red light using LED lamps. Sci Hortic-Amsterdam. 2020;268:109371. doi: 10.1016/j.scienta.2020.109371.

[21] Du J , Liu Y , Xu Z , Hao X , Song H , Guo M , Zhang Y , Meng L . Transcriptomic analysis reveals light quality response and systemic nutrient allocation under monochromatic light environments in nicotiana tabacum. BMC Plant Biol. 2025;25:1750. doi: 10.1186/s12870-025-07974-w. 41421981 PMC12750675

[22] Ning W , Yang Y , Chen W , Li R , Cao M , Luo J . Effect of light combination on the characteristics of dissolved organic matter and chemical forms of cd in the rhizosphere of arabidopsis thaliana involved in phytoremediation. Ecotox Environ Safe. 2022;231:113212. doi: 10.1016/j.ecoenv.2022.113212. 35065501

[23] Gawande V , Priya , Raut D , Rai S , Beese S , Singh BV , Agnihotri N . Artificial light spectra and its impact on plant physiological processes and secondary metabolism. Int J Plant Soil Sci. 2023;35:2060–2070. doi: 10.9734/ijpss/2023/v35i183492.

[24] Izzo LG , Mickens MA , Aronne G , Gómez C . Spectral effects of blue and red light on growth, anatomy, and physiology of lettuce. Physiol Plantarum. 2021;172:2191–2202. doi: 10.1111/ppl.13395. 33715155

[25] Song Z , Lin C , Xing P , Fen Y , Jin H , Zhou C , Gu YQ , Wang J , Li XA . High-quality reference genome sequence of *salvia miltiorrhiza* provides insights into tanshinone synthesis in its red rhizomes. Plant Genome. 2020;13:e20041. doi: 10.1002/tpg2.20041. 33217202 PMC12807052

[26] Tian T , Liu Y , Yan H , You Q , Yi X , Du Z , Xu W , Su Z . agriGO v2.0: a GO analysis toolkit for the agricultural community, 2017 update. Nucleic Acids Res. 2017;45:W122–W129. doi: 10.1093/nar/gkx382. 28472432 PMC5793732

[27] Chen C , Chen H , Zhang Y , Thomas HR , Frank MH , He Y , Xia R . TBtools: an integrative toolkit developed for interactive analyses of big biological data. Mol Plant. 2020;13:1194–1202. doi: 10.1016/j.molp.2020.06.009. 32585190

[28] Li Y , Chen Y , Zhou L , You S , Deng H , Chen Y , Alseekh S , Yuan Y , Fu R , Zhang Z , et al. MicroTom metabolic network: rewiring tomato metabolic regulatory network throughout the growth cycle. Mol Plant. 2020;13:1203–1218. doi: 10.1016/j.molp.2020.06.005. 32561360

[29] Ma Y , Zhang J , Liang K , Huang A , Liu J , Li W , Sun B , Zhao Y . Overexpression of *osmybs1* affect leaf morphology, photosynthesis, and agronomic performance in rice. Front Plant Sci. 2025;16. doi: 10.3389/fpls.2025.1653514. PMC12518331 41098921

[30] Shi Y , Ke X , Yang X , Liu Y , Hou X . Plants response to light stress. J Genet Genomics. 2022;49:735–747. doi: 10.1016/j.jgg.2022.04.017. 35580763

[31] Iqbal Z , Iqbal MS , Hashem A , Abd_Allah EF , Ansari MI . Plant defense responses to biotic stress and its interplay with fluctuating Dark/Light conditions. Front Plant Sci. 2021;12. doi: 10.3389/fpls.2021.631810. PMC7982811 33763093

[32] Yang B , Tang J , Yu Z , Khare T , Srivastav A , Datir S , Kumar V . Light stress responses and prospects for engineering light stress tolerance in crop plants. J Plant Growth Regul. 2019;38:1489–1506. doi: 10.1007/s00344-019-09951-8.

[33] Yousef AF , Ali MM , Rizwan HM , Tadda SA , Kalaji HM , Yang H , Ahmed MAA , Wróbel J , Xu Y , Chen F . Photosynthetic apparatus performance of tomato seedlings grown under various combinations of LED illumination. PLoS One. 2021;16:e0249373. doi: 10.1371/journal.pone.0249373. 33858008 PMC8049771

[34] de Wit M , Spoel SH , Sanchez-Perez GF , Gommers CMM , Pieterse CMJ , Voesenek LACJ , Pierik R . Perception of low red:far-red ratio compromises both salicylic acid- and jasmonic acid-dependent pathogen defences in. Arabidopsis Plant J. 2013;75:90–103. doi: 10.1111/tpj.12203. 23578319

[35] Zhu M , Geng S , Chakravorty D , Guan Q , Chen S , Assmann SM . Metabolomics of red-light-induced stomatal opening in arabidopsis thaliana: coupling with abscisic acid and jasmonic acid metabolism. Plant J. 2020;101:1331–1348. doi: 10.1111/tpj.14594. 31677315

[36] Nagata M , Yamamoto N , Shigeyama T , Terasawa Y , Anai T , Sakai T , Inada S , Arima S , Hashiguchi M , Akashi R , et al. Hirsch, A. M.; suzuki, A., Red/Far red light controls arbuscular mycorrhizal colonization via Jasmonic acid and strigolactone signaling. Plant Cell Physiol. 2015;56:2100–2109. 26412782 10.1093/pcp/pcv135

[37] Chen Y , Shi L , Xu Q , Zhang C , Wang L , Li W . Light signal transduction networks regulating phenylpropanoid, terpenoid and alkaloid biosynthesis in horticultural plants. J Plant Physiol. 2026;317:154681. doi: 10.1016/j.jplph.2025.154681. 41455240

[38] Ge Y , Chen Y , Li C , Zhao J , Wei M , Li X , Yang S , Mi Y . Effect of sodium nitroprusside treatment on shikimate and phenylpropanoid pathways of apple fruit. Food Chem. 2019;290:263–269. doi: 10.1016/j.foodchem.2019.04.010. 31000046

[39] Xie Z , Kapteyn J , Gang DR . A systems biology investigation of the MEP/terpenoid and shikimate/phenylpropanoid pathways points to multiple levels of metabolic control in sweet basil glandular trichomes. Plant J. 2008;54:349–361. doi: 10.1111/j.1365-313X.2008.03429.x. 18248593

[40] Zha L , Wei S , Huang D , Zhang J . Multi-omics analyses of lettuce (*lactuca sativa*) reveals primary metabolism reorganization supporting distinct features of secondary metabolism induced by supplementing UV-A radiation. J Agricul Food Chem. 2024;72:15498–15511. doi: 10.1021/acs.jafc.4c00394. 38950542

[41] Fan X , Ge A-H , Qi S , Guan Y , Wang R , Yu N , Wang E . Root exudates and microbial metabolites: signals and nutrients in plant-microbe interactions. Sci China Life Sci. 2025;68:2290–2302. doi: 10.1007/s11427-024-2876-0. 40080268

[42] Afzal MY , Das BK , Valappil VT , Scaria J , Brözel VS . Root exudate compounds change the bacterial community in bulk soil. Rhizosphere. 2024;30:100885. doi: 10.1016/j.rhisph.2024.100885.

[43] Rawat A , Srivastava SK , Tiwary CS , Gupta AK . An LED-driven Hematite/Bi4O5I2 nanocomposite as an S-scheme heterojunction photocatalyst for efficient degradation of phenolic compounds in real wastewater. J Mater Chem A. 2025;13:1271–1286. doi: 10.1039/D4TA07324J.

[44] Ban F , Ye C , Yang S , Li M . Degradation of phenol by UV light-assisted electrocatalytic treatment. J Iran Chem Soc. 2023;20:2031–2041. doi: 10.1007/s13738-023-02821-x.

[45] Yanlong C , Kejian Y , Yin Y , Yuhan Z , Huizi M , Cui L , Zhonghui L , Ziru P , Fan C , Jiangtao Y , et al. Reductive soil disinfestation attenuates antibiotic resistance genes in greenhouse vegetable soils. J Hazard Mater. 2021;420:126632. 34293692 10.1016/j.jhazmat.2021.126632

[46] Hoque MN , Rana ML , Gilman MAA , Pramanik PK , Islam MS , Punom SA , Rahman R , Hassan J , Rahman MS , Ramasamy S , et al. Rooftop and surface garden soils in Bangladesh harbor diverse resistome profiles. Environ Monit Assess. 2026;198:396. doi: 10.1007/s10661-026-15240-1. 41920399

[47] Apollon W . Microbial fuel cells as modulators of plant growth and secondary metabolism: a mechanistic review. Bioresour Technol Rep. 2026;34:102726. doi: 10.1016/j.biteb.2026.102726.

[48] Ye F , Jiang M , Zhang P , Liu L , Liu S , Zhao C , Li X . Exogenous melatonin reprograms the rhizosphere microbial community to modulate the responses of barley to drought stress. Int J Mol Sci. 2022;23:9665. doi: 10.3390/ijms23179665. 36077064 PMC9456345

[49] Chevrette MG , Thomas CS , Hurley A , Rosario-Meléndez N , Sankaran K , Tu Y , Hall A , Magesh S , Handelsman J . Microbiome composition modulates secondary metabolism in a multispecies bacterial community. P NATL ACAD SCI USA. 2022;119:e2212930119. doi: 10.1073/pnas.2212930119. PMC9586298 36215464

