# Supplementary material for: Monochromatic light reprograms transcription, metabolism, and rhizosphere microbial communities in Salvia miltiorrhiza
Source: Plant Signal Behav. 2026 Jun 15;21(1):2686334. doi: 10.1080/15592324.2026.2686334 (PMC13274158; doi:10.1080/15592324.2026.2686334)
Supplement: Supplemental Figures-clean.docx [file KPSB_A_2686334_SM2205.docx]

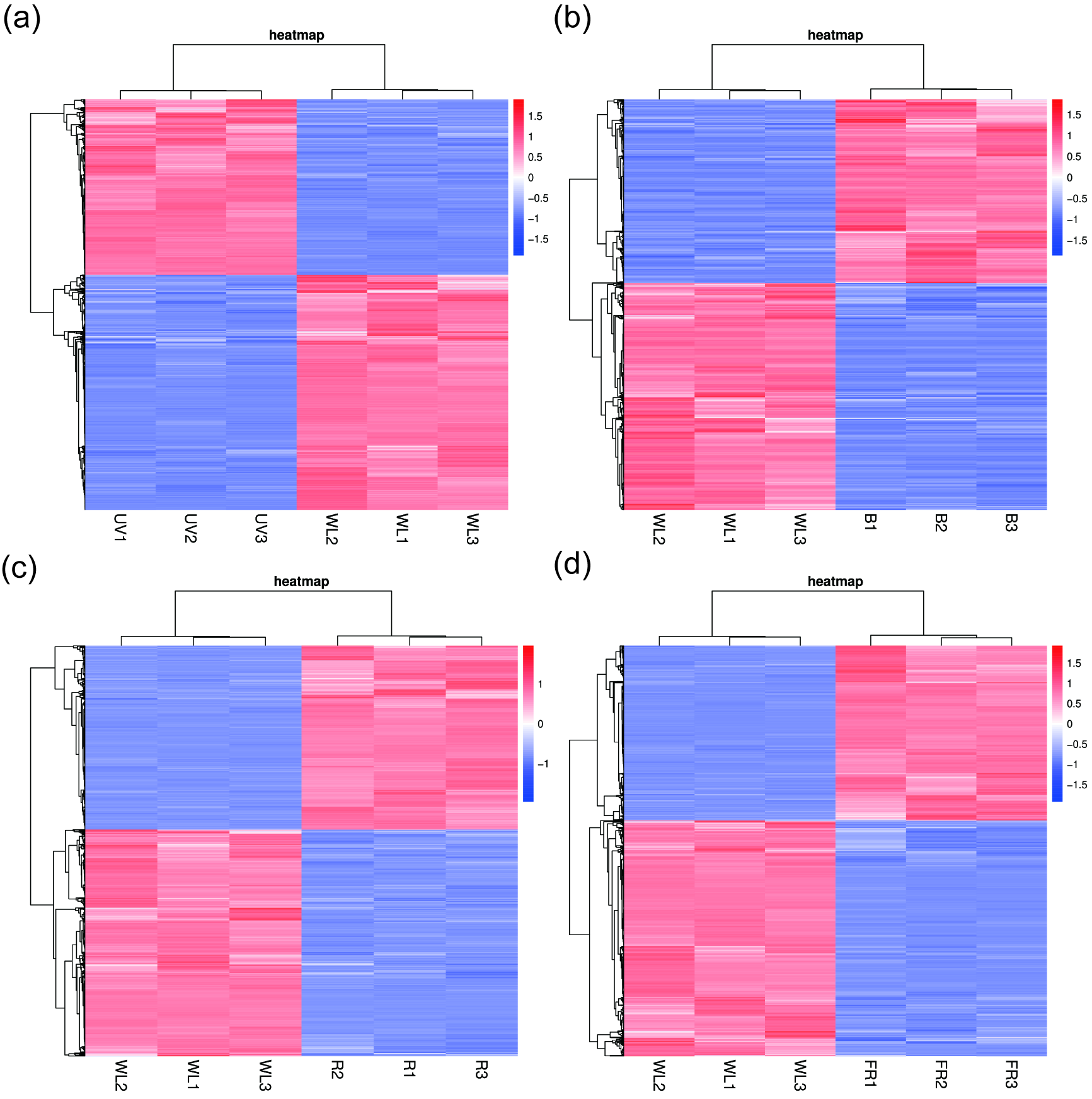


**Supplemental Figure 1.** Heatmaps of differentially expressed genes (DEGs) in *S. miltiorrhiza* under different monochromatic light cultures. (**a**) WL_UV, (**b**) WL_B, (**c**) WL_R, (**d**)WL_FR. Each column represents a biological replicate, and each row represents a DEG. Color scale represents expression values .

**
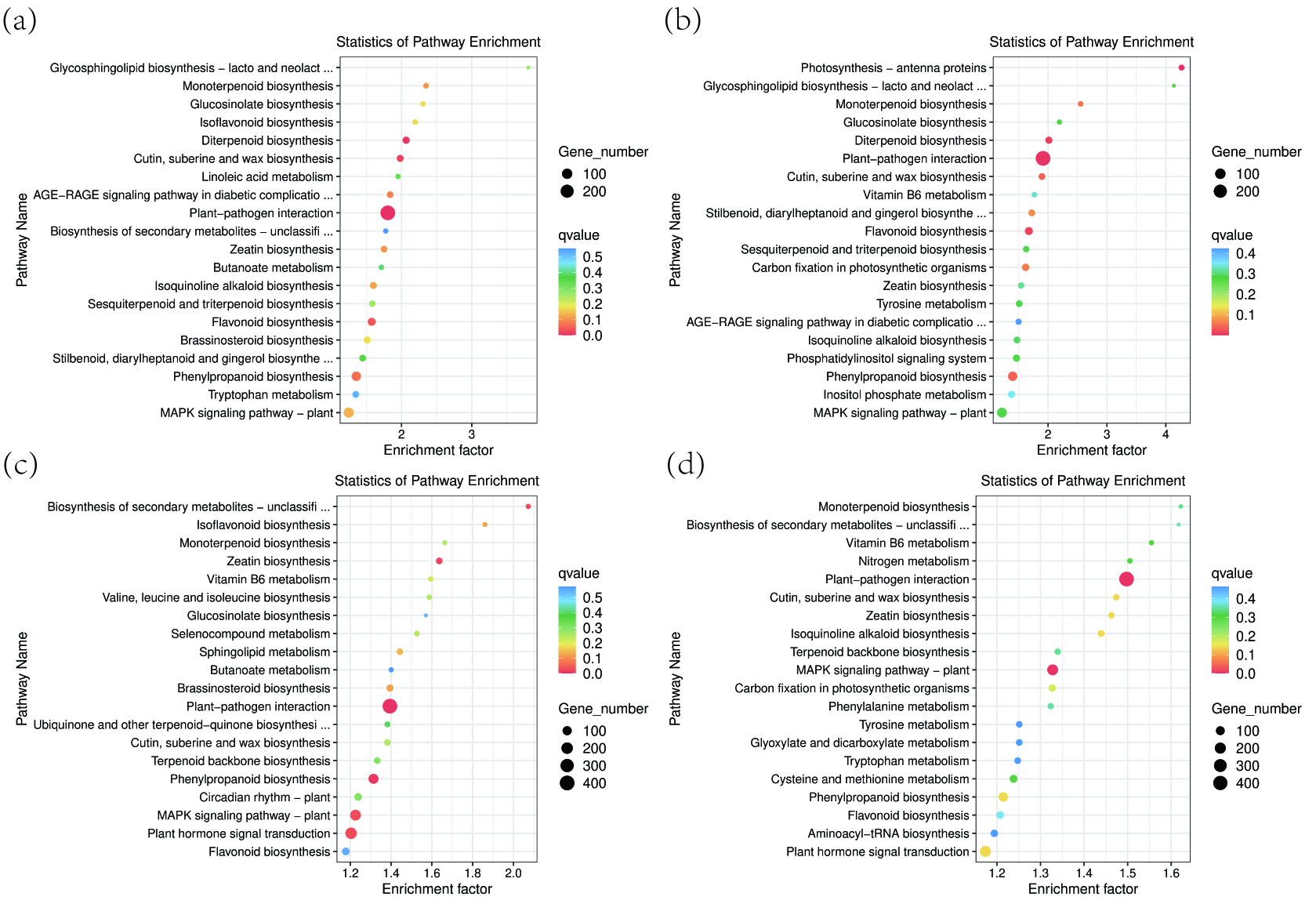
**

**Supplemental Figure 2.** KEGG pathway enrichment analysis of DEGs in different monochromatic light cultures. (**a**) WL_UV, (**b**) WL_B, (**c**) WL_R, (**d**) WL_FR. Only the top 10 most significantly enriched pathways are shown for each group. Dot size represents the number of DEGs, and color represents the p-value.

**
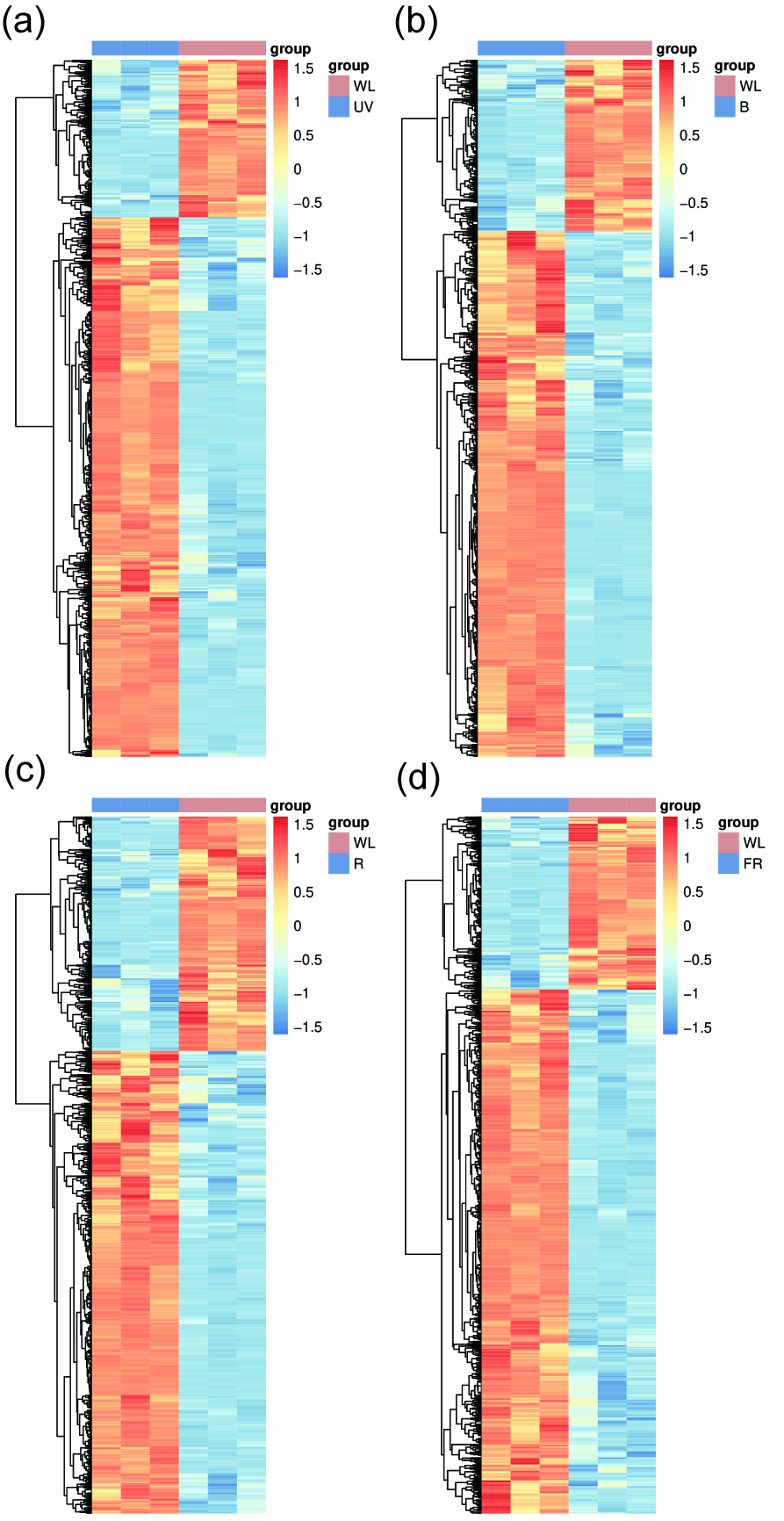
**

**Supplemental Figure 3.** Heatmaps of differentially expressed metabolites (DEMs) in *S. miltiorrhiza* under different monochromatic light cultures. (**a**) WL_UV, (**b**) WL_B, (**c**) WL_R, (**d**) WL_FR. Each column represents a biological replicate, and each row represents a DEM. Color scale represents scaled metabolite abundance.


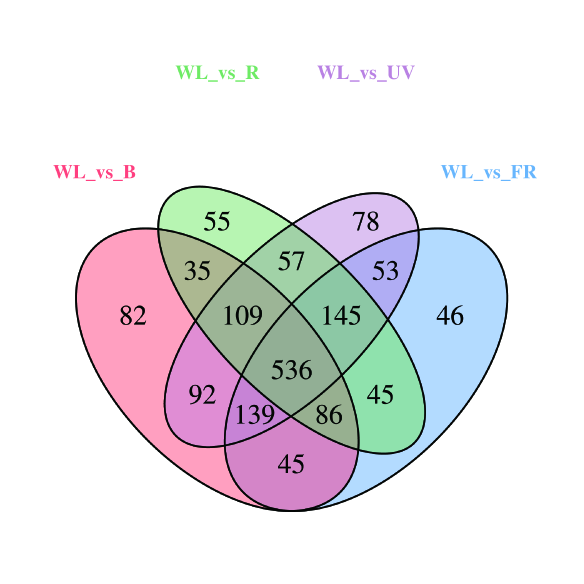


**Supplemental Figure 4.** Venn diagram of DEMs across all treatment groups. The diagram shows the number of DEMs unique to each treatment group and those shared among groups .


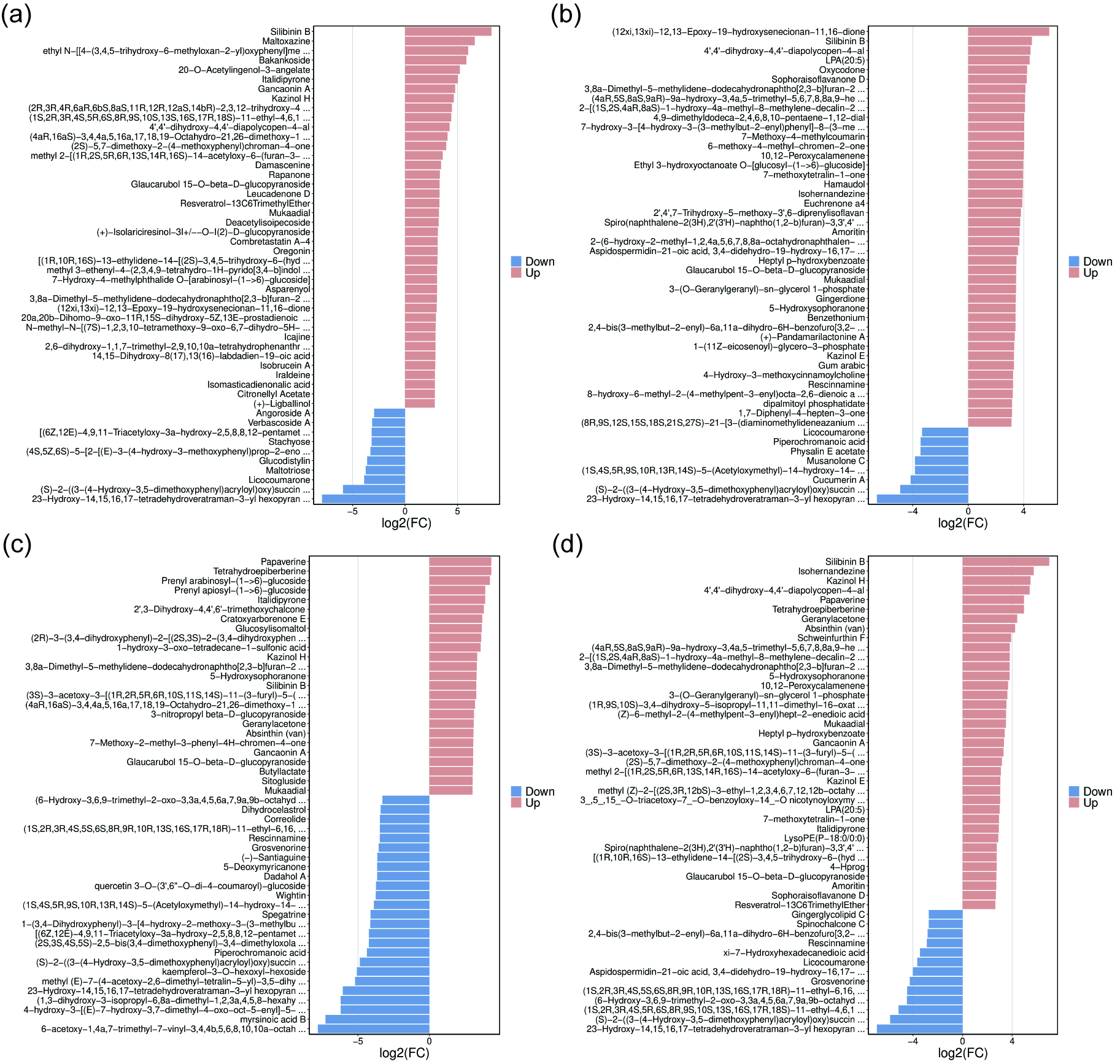


**Supplemental Figure 5.** Volcano plots of DEMs in *S. miltiorrhiza* under different monochromatic light cultures. (**a**) WL_UV, (**b**) WL_B, (**c**) WL_R, (**d**) WL_FR. Significantly upregulated metabolites (red), significantly downregulated metabolites (blue), and non-significant metabolites (blue) are shown. The names of representative significantly changed metabolites are labeled.


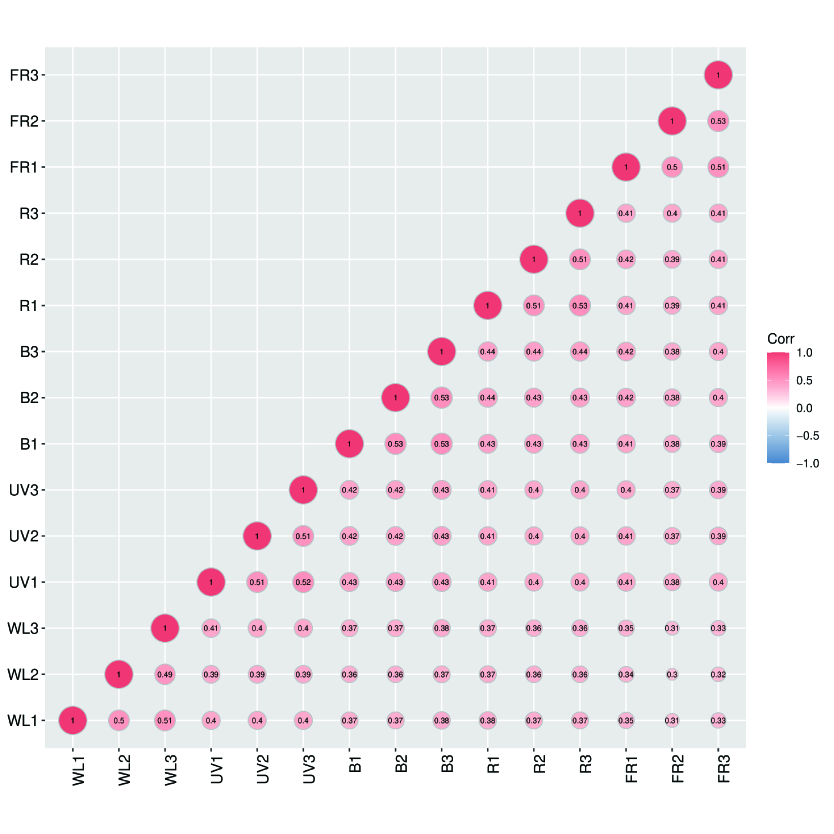


**Supplemental Figure 6.** Correlation analysis of metagenomic samples from *S. miltiorrhiza* rhizosphere under different monochromatic light conditions. A heatmap showing the Spearman correlation coefficients among all samples. Red indicates high positive correlation, blue indicates low correlation.


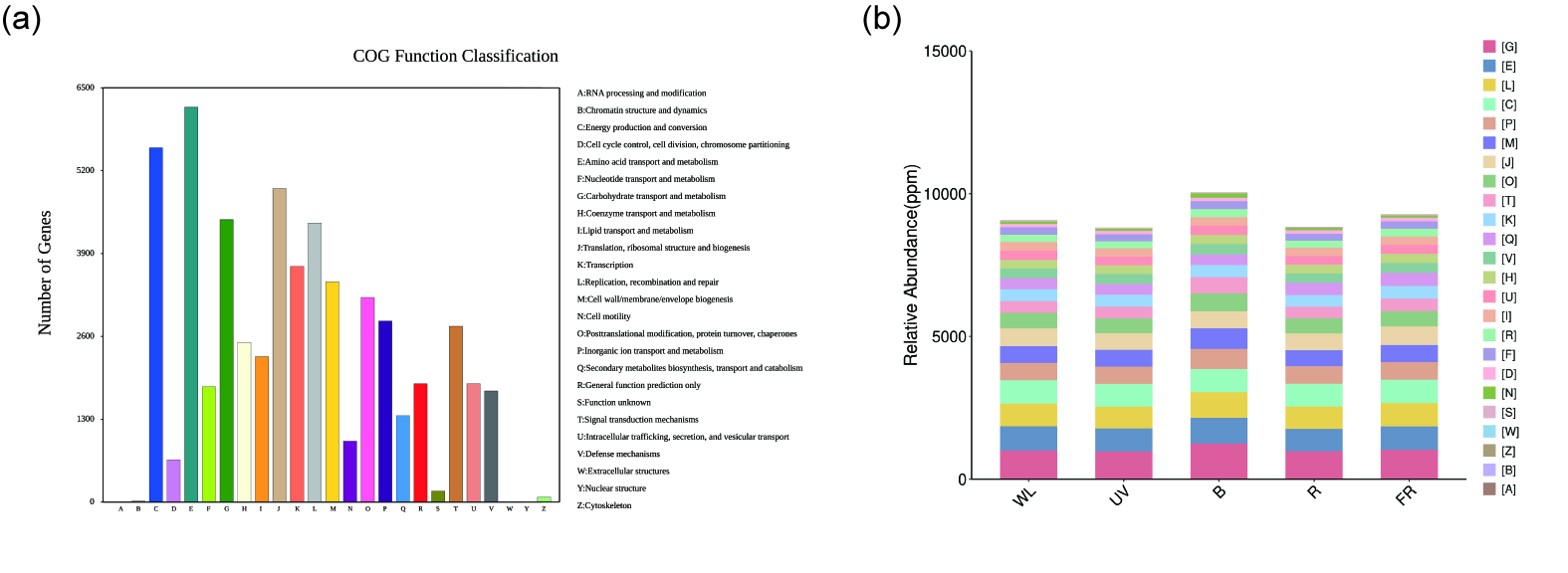


**Supplemental Figure 7.** COG analysis of metagenomic data. (**a**) Number of microbial genes assigned to each COG functional category across different light treatments. (**b**) Relative abundance of each COG functional category across different light treatments.


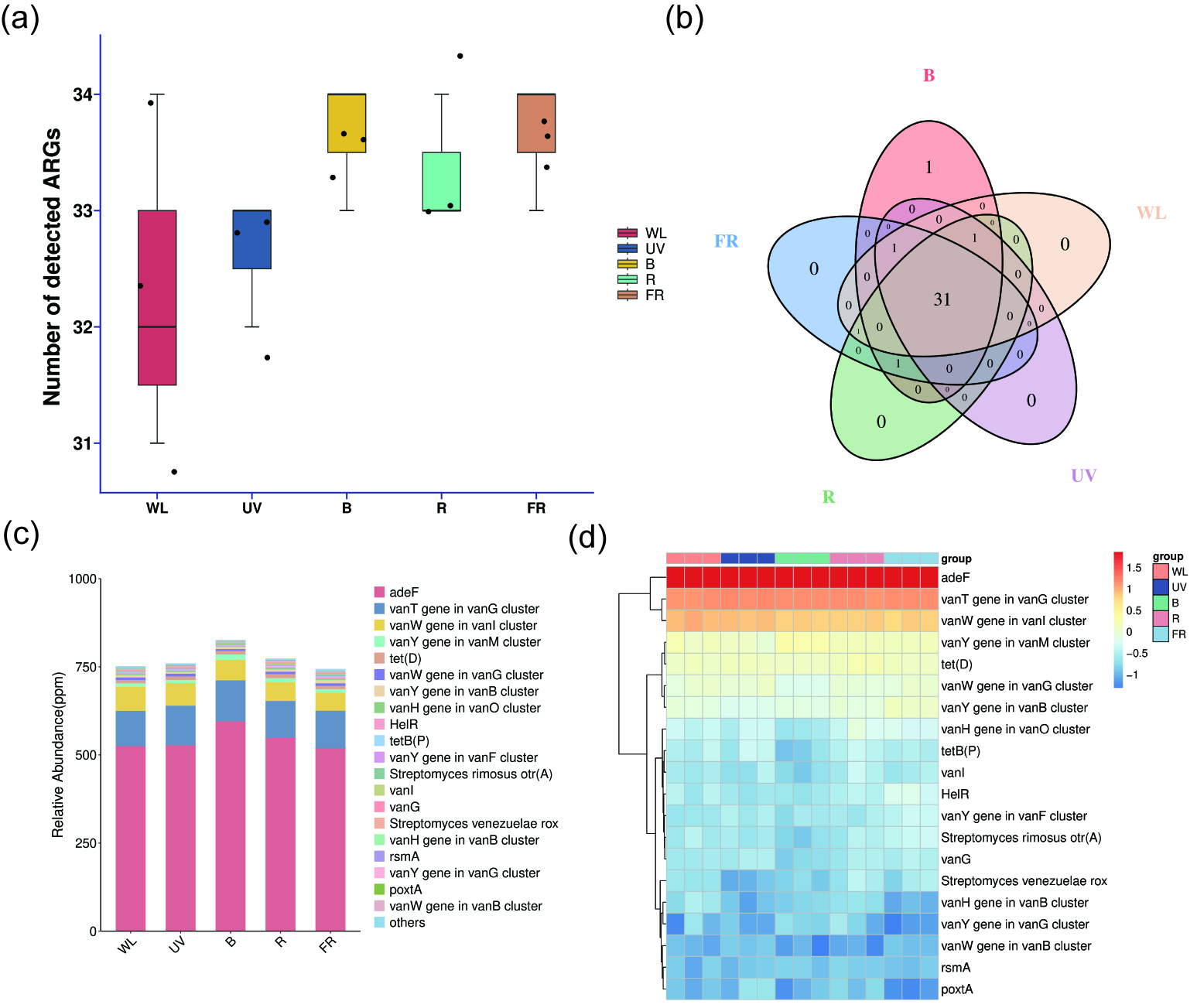


**Supplemental Figure 8.** CARD analysis of metagenomic data. (**a**) Number of detected antibiotic resistance genes (ARGs) across different light treatments. (**b**) Venn diagram of detected ARGs across all treatment groups. (**c**) Relative abundance of the adeF ARG across different light treatments. (**d**) Heatmap showing the relative abundance of all detected ARGs across different light treatments. Color scale represents row-scaled Z-scores.


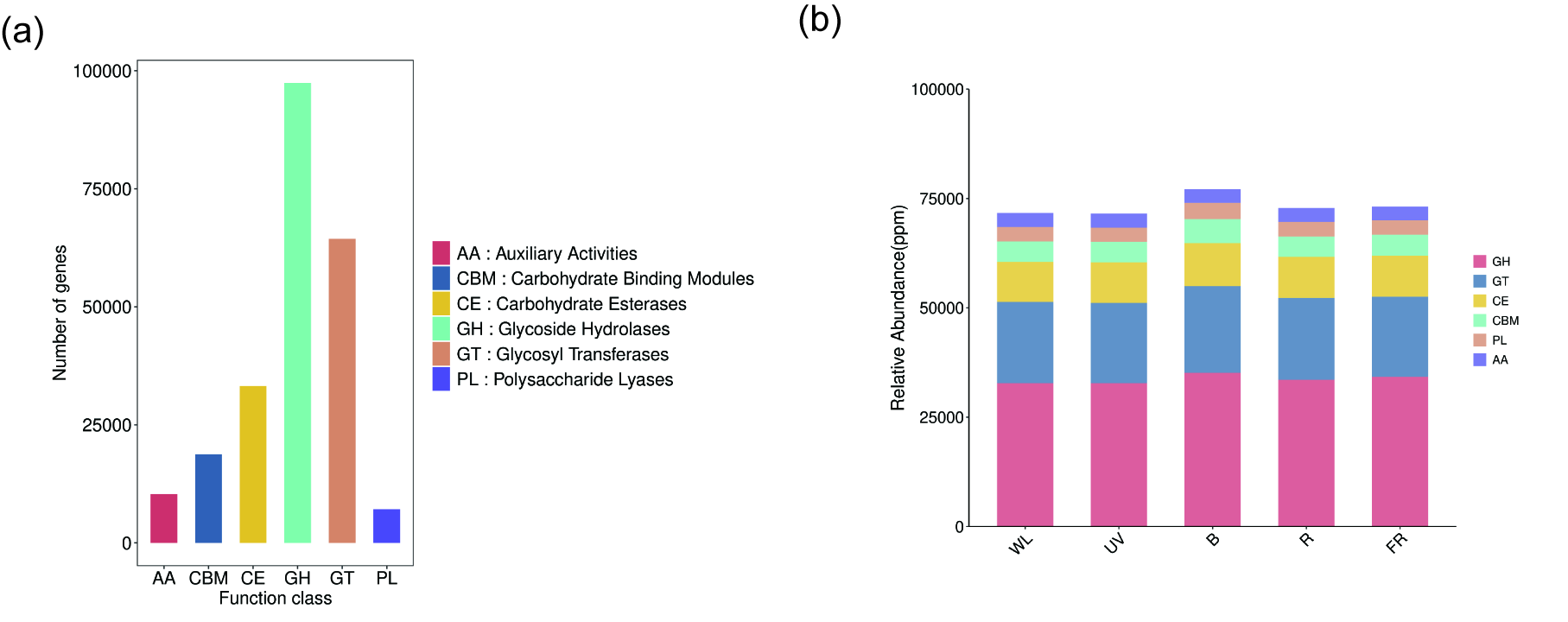


**Supplemental Figure 9.** CAZy analysis of metagenomic data. (**a**) Number of microbial genes assigned to each CAZy functional class (AA: auxiliary activities; CBM: carbohydrate-binding modules; CE: carbohydrate esterases; GH: glycoside hydrolases; GT: glycosyl transferases; PL: polysaccharide lyases) across different light treatments. (**b**) Relative abundance of each CAZy functional class across different light treatments.


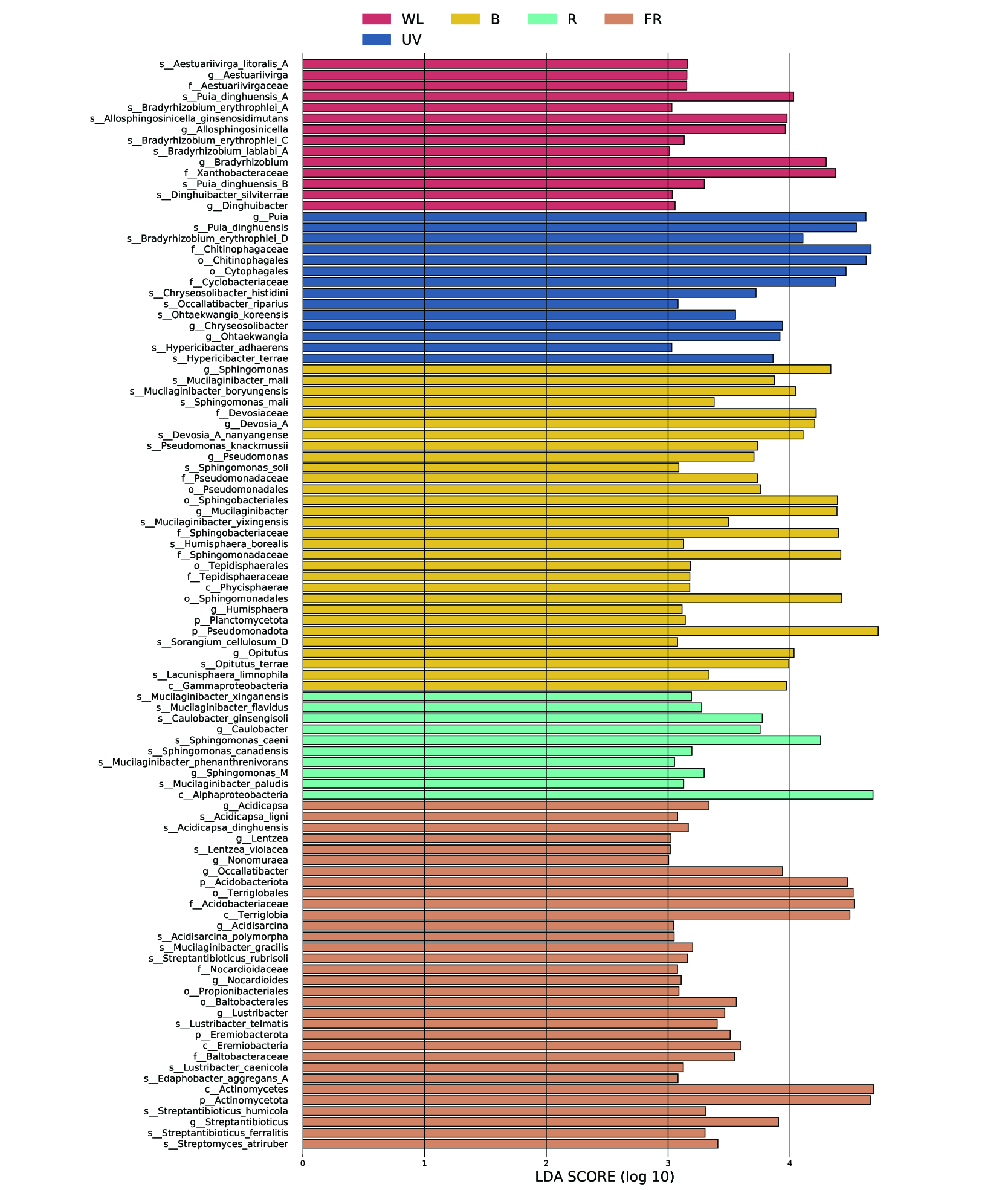


**Supplemental Figure 10.** Linear discriminant analysis Effect Size (LEfSe) of differentially abundant microbial taxa under different monochromatic light conditions. Histogram of LDA scores (log_10_) for taxa with significant differential abundance (LDA score > 2, p < 0.05). The color indicates the treatment group in which the taxon is enriched (WL, UV, B, R, FR). The taxa with the highest LDA score for each group are labeled.


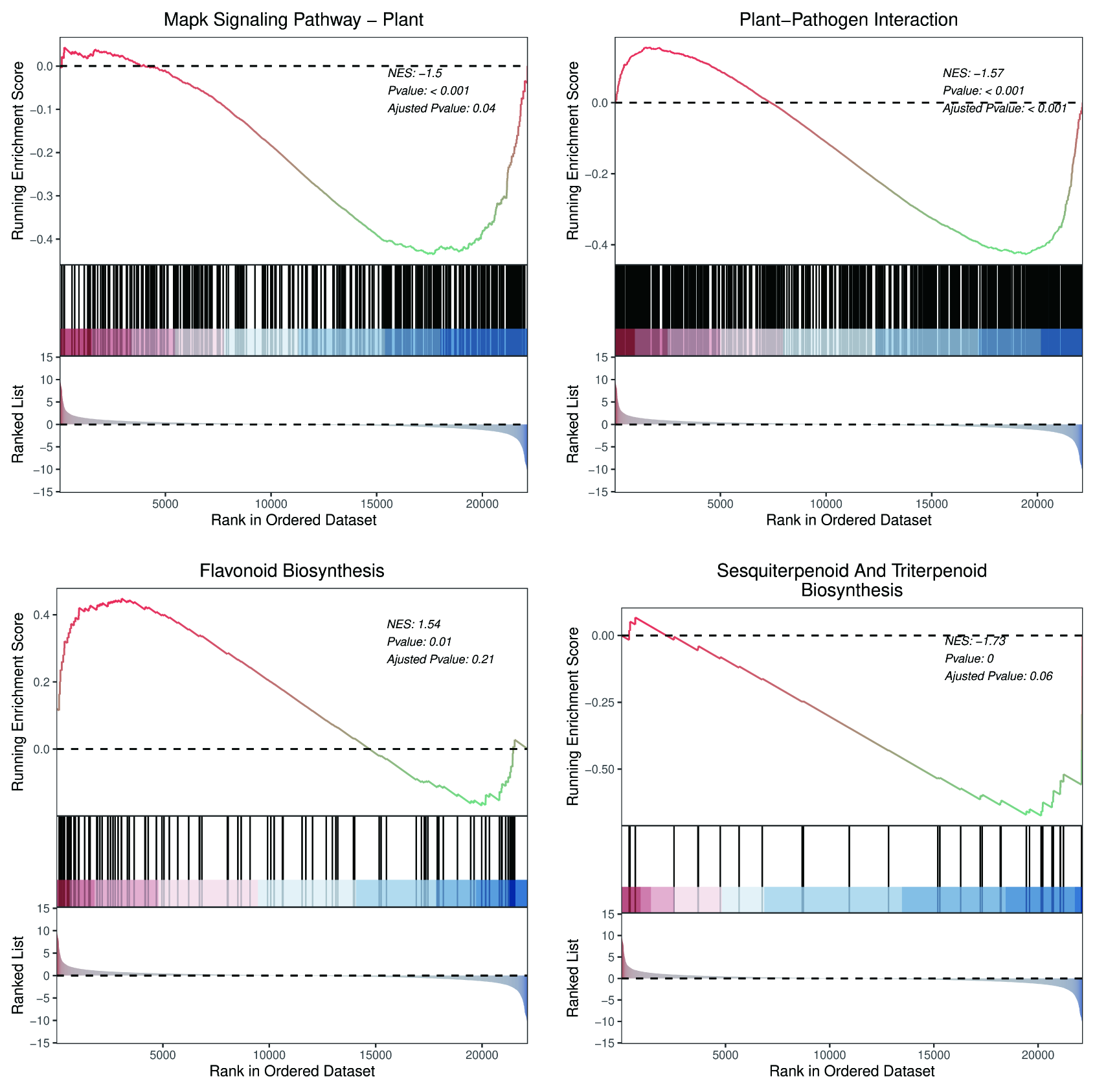


**Supplemental Figure 11.** Gene Set Enrichment Analysis (GSEA) analysis for KEGG maps in WL_UV group. Top: Running enrichment score (ES) curve; the maximum deviation from zero defines the ES. Middle: Barcode plot showing positions of gene set members in the ranked list (sorted by log₂FC). Bottom: Ranked gene metric distribution; red (high in group B), blue (high in group A), and area chart for signal-to-noise ratio. ES measures cumulative enrichment; NES is the normalized ES. Positive NES: upregulation tendency; negative NES: downregulation tendency; NES near 0: no significant enrichment.


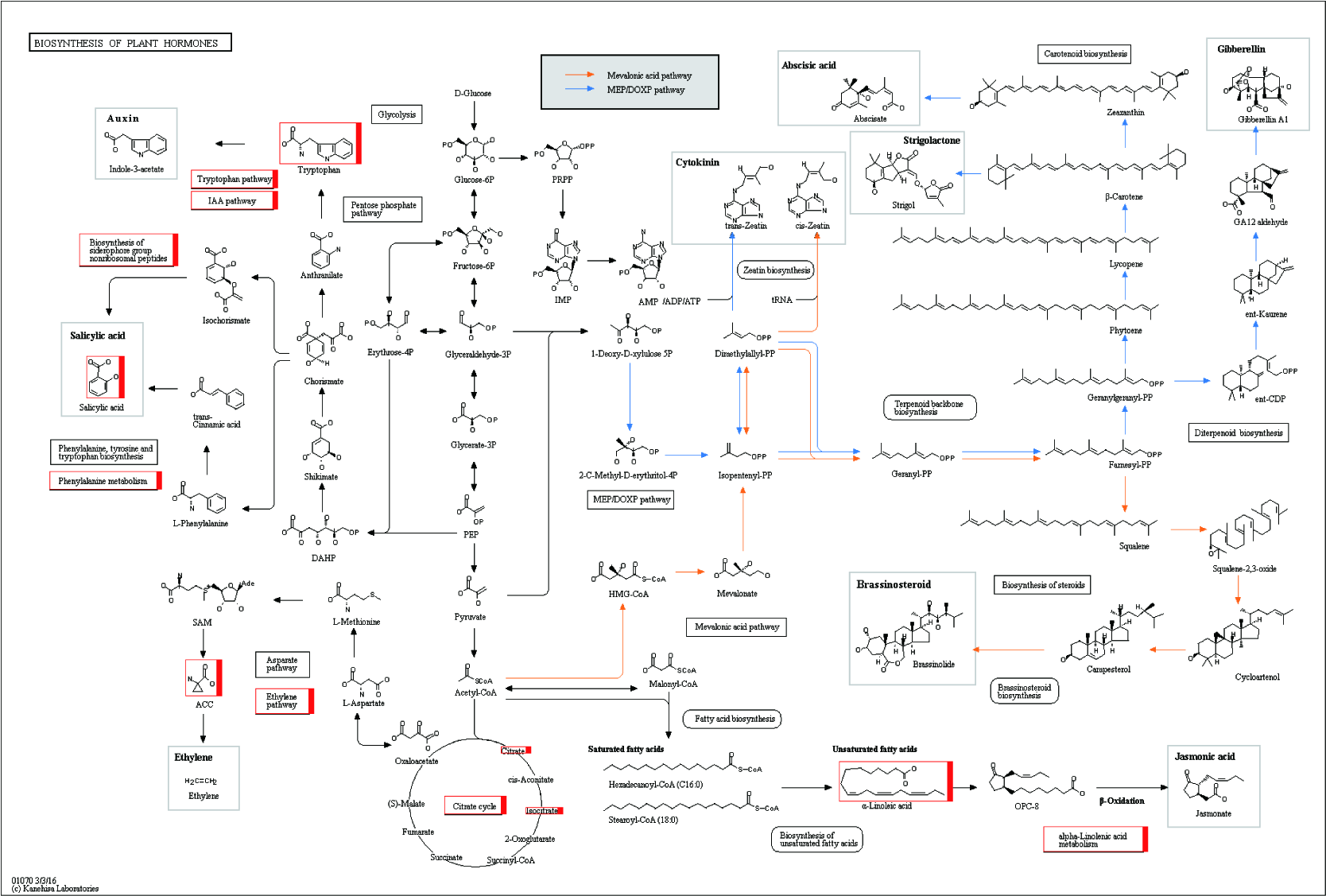


**Supplemental Figure 12.** DEMs in biosynthesis of pant hormones pathway in WL_UV group. Red: up-regulated metabolites.


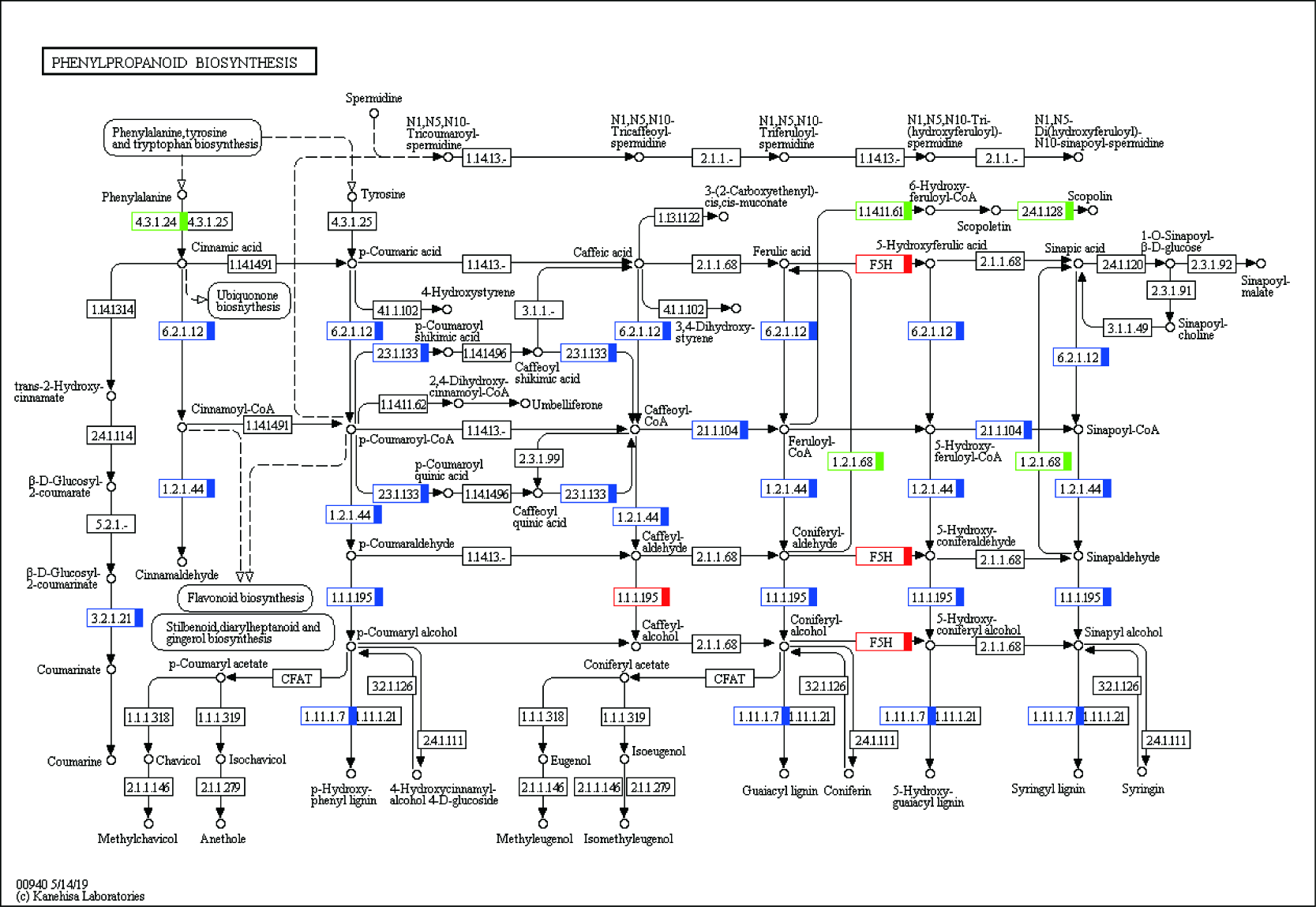


**Supplemental Figure 13.** DEGs in Phenylpropanoid biosynthesis pathway in WL_UV group. Red: up-regulated genes, Green: down-regulated genes, Blue: up/down-regulated genes.


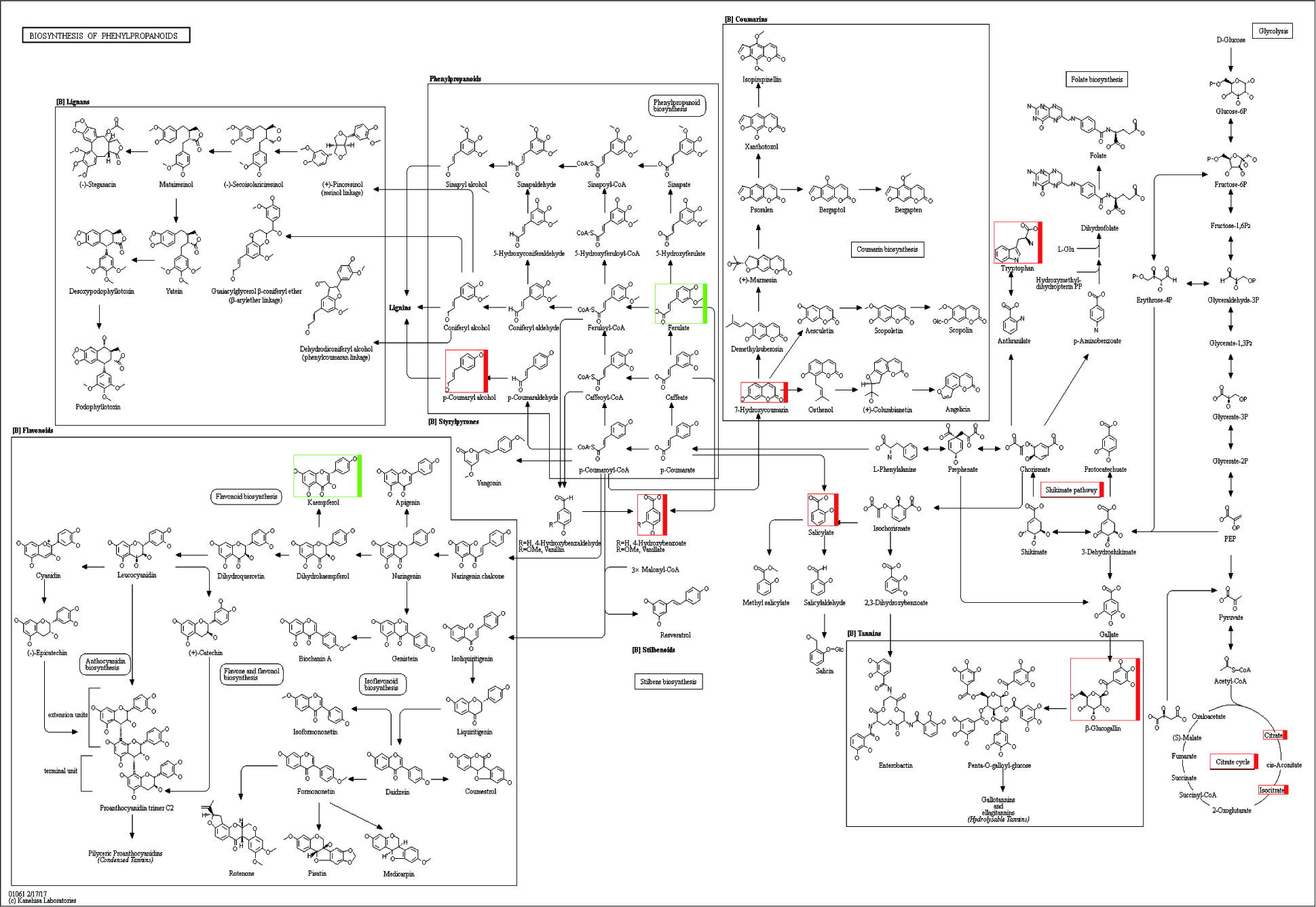


**Supplemental Figure 14.** DEMs in Phenylpropanoid biosynthesis pathway in WL_UV group. Red: up-regulated metabolites, Green: down-regulated metabolites.
